# DeSIDE-DDI: interpretable prediction of drug-drug interactions using drug-induced gene expressions

**DOI:** 10.1186/s13321-022-00589-5

**Published:** 2022-03-04

**Authors:** Eunyoung Kim, Hojung Nam

**Affiliations:** School of Electrical Engineering and Computer Science, Gwangju Institute of Science and Technology (GIST), Buk-gu, Gwangju, 61005 Republic of Korea

**Keywords:** Drug-drug interaction, Polypharmacy side effects, In silico prediction, Deep learning

## Abstract

Adverse drug-drug interaction (DDI) is a major concern to polypharmacy due to its unexpected adverse side effects and must be identified at an early stage of drug discovery and development. Many computational methods have been proposed for this purpose, but most require specific types of information, or they have less concern in interpretation on underlying genes. We propose a deep learning-based framework for DDI prediction with drug-induced gene expression signatures so that the model can provide the expression level of interpretability for DDIs. The model engineers dynamic drug features using a gating mechanism that mimics the co-administration effects by imposing attention to genes. Also, each side-effect is projected into a latent space through translating embedding. As a result, the model achieved an AUC of 0.889 and an AUPR of 0.915 in unseen interaction prediction, which is competitively very accurate and outperforms other state-of-the-art methods. Furthermore, it can predict potential DDIs with new compounds not used in training. In conclusion, using drug-induced gene expression signatures followed by gating and translating embedding can increase DDI prediction accuracy while providing model interpretability. The source code is available on GitHub (https://github.com/GIST-CSBL/DeSIDE-DDI).

**Supplementary Information:**

The online version contains supplementary material available at 10.1186/s13321-022-00589-5.

## Introduction

Polypharmacy has resulted in an increasing number of patients taking multiple drugs simultaneously. This can be problematic because drug interactions can alter the intended responses. In some cases, it leads to unexpected side effects or decreased clinical efficacy. This phenomenon is commonly referred to as a drug-drug interaction (DDI). Identifying potential DDIs in advance is crucial because the most vulnerable people in society (e.g., older people and multimorbid patients) are the primary targets of polypharmacy.

DDIs are generally found through experimentation. However, in vitro and in vivo identification of DDIs are largely infeasible, owing to patient safety and ethical considerations that increase time and costs. Furthermore, major polypharmacy side effects are difficult to identify from small trials and cohorts [1]. Therefore, computational approaches have been developed. Many computational approaches use datasets retrieved from past studies, electronic health records, and social media [2–4]. Similarity-based and network-based approaches were commonly used in predicting DDIs. Contemporary similarity-based approaches assume that similar drugs may have similar interactions. Such similarities are obtained based on drug structures, targets, ontologies, and side effects, which are utilized as features for machine-learning training [5–9]. In the case of network-based approaches, novel interactions are inferred via network analyses. Interactions are predicted using label propagation or newly calculated features [10]. Additionally, various graph-embedding algorithms have been proposed using graph analysis, showing potentially effective network-based features [11–18].

Recently, deep learning has demonstrated outstanding performance in various domains, including DDI prediction tasks. Deep neural networks have been applied to prediction models using various drug features. Previously, DeepDDI, which comprises eight hidden layers, was developed to consider the structural similarities of two drugs [19]. Another model (i.e., DDIMDL) was constructed based on the similarity assumption, and its architecture is a multimodal deep neural network that comprises separate models of each similarity [20]. Unlike traditional approaches that predict the possibility of interactions, two proposed models are multi-class classifiers that indicate interaction types presenting the ability of predicting specific side effect types. Additionally, graph neural networks (GNNs) have improved the performance of graph- or node-embedding approaches. Decagon operated graph convolutional networks on a heterogeneous network comprised of DDIs, protein–protein interactions (PPIs), and drug-target interactions [21]. The model updates node features in consideration of side-effect-related neighbor nodes and further predicts whether drug combinations have interactions. GNNs were applied to predict DDIs with consideration of indirect interactions and to represent compound structures [22–25]. Moreover, GNNs have been employed on both compounds and interactions networks achieving good performance [26]. Recently, the National Institutes of Health conducted the Library of Integrated Network-based Cellular Signatures (LINCS) L1000 project, which provides drug-induced gene expression profile information under various conditions [27]. Transcriptome-level information is provided that describes detailed drug responses. A recent study proposed an artificial neural network (ANN) that uses L1000 data [28] for gene expression, gene ontology, and compound fingerprint information as features. Their proposal includes a multi-label classification ANN model with three simple hidden layers. However, the performance of the model is relatively poor compared with previous deep-learning models, indicating the need for a more sophisticated method of handling transcriptome signals.

**Fig. 1:**
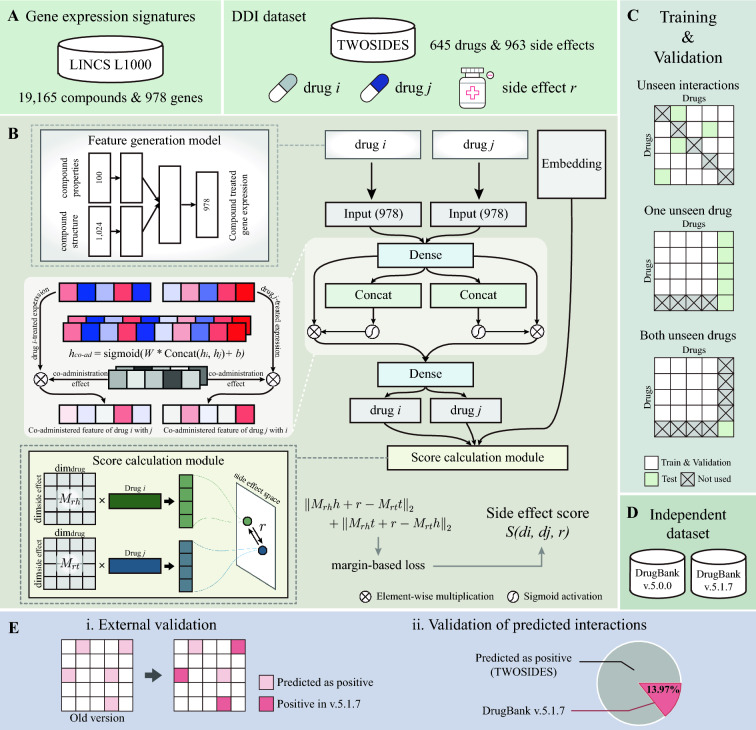
Overview of the drug-drug interaction (DDI) prediction model. **A** Datasets are preprocessed for each feature generation model and DDI prediction model. **B** Given drug-pair and side-effect data, each drug feature is obtained using a feature generation model and a side effect is embedded to latent space; then, drugs pass through neural networks having gated linear units to consider the pair information. Finally, using latent representations of drugs and the side effect, the side-effect score is calculated. **C** The model is evaluated considering three DDI cases. **D**, **E** The independent dataset from DrugBank is used for external validation

Notwithstanding the numerous extant studies, many problems are waiting to be solved. First, most studies utilized structures and/or properties of the drug compound while less concerning interpretation on molecular levels of drug response signals. Because the prediction models only used features originating from compounds, the models cannot capture the characteristics of the DDI mechanism derived from drug treatments. Second, predicting the interactions of new compounds not used in training remains a significant challenge. To address these issues, we developed a deep-learning strategy for interpretable prediction of DDIs that leverages drug-induced gene expression signatures (DeSIDE-DDI). As shown in Fig. 1, we designed to predict triplets that represent the relationship between one drug pair and one side effect. When representing drugs, we use drug-induced gene expressions to account for how drugs affect gene expression changes. To allow the utilization of all possible compounds, we develop a feature generation model. A gate unit is then applied to drug features to mimic drug co-administration effects by imposing attention to important genes, which engineers dynamic features of one drug when combined with others. Then, each feature is projected to each side-effect space in the score calculation module. The robustness of our models was verified through several rigorous tests. Overall, the proposed model was confirmed to be an accurate and interpretable DDI prediction model for predicting potential side-effects among drug combinations.

## Materials and methods

### Datasets

Our model consists of two parts: one of feature generation and another of DDI prediction. The feature generation model generates drug-induced gene expression, which uses compound structures and property information for inputs and outputs predicted gene expressions. The gold-standard dataset of the model is the LINCS L1000 database [27]. These data contain gene expression profiles of multiple cell lines treated with thousands of chemicals at different time points and doses. Among the different levels of L1000 data, we used the level-5 data, which provide differential gene expression signatures. First, we extracted signatures of 978 landmark genes that were directly measured for confidence. Then, because each gene expression signature of a compound was taken under various sets of experimental conditions, we used “Signature Strength” (SS), which is the representative signature of a compound showing the most significant change [29]. Finally, we selected organic compounds and obtained gene expression signatures of 19,156 compounds (Table 1).

**Table 1:** LINCS L1000 data statistics of feature generation model

	Raw signature information (drug-induced only)	Processed
#Compounds	20,413	19,156
#Cell lines	72	58
#Genes	12,328	978
#Response signatures	251M	18M

To train the DDI prediction model, two DDI datasets were used in our study: TWOSIDES and DrugBank [30, 31]. The TWOSIDES database provides processed polypharmacy side effects retrieved from the US Food and Drug Administration adverse reporting systems. We downloaded BioSNAP, which is an additionally refined dataset of TWOSIDES [32]. We filtered out uncommon side effects from the raw data that occurred with less than 500 drug combinations, as performed by previous studies. Finally, we obtained 4,576,287 interactions between 63,472 drug combinations and 963 side effects. Also, DrugBank data were used for external validation. We downloaded old and new versions of DrugBank (v. 5.0.0 and 5.1.7). Only DDIs were extracted from the raw extended markup-language file, each containing 33,497 interactions among 1129 drugs and 782,405 interactions among 2616 drugs. The details of data statistics are listed in Table 2.

**Table 2:** Data statistics of DDI prediction model

Database	#Drugs	#Drug combinations	#DDI types	#Triplets
TWOSIDES	645	63,472	963	4,576,287
DrugBank (ver5.0.0)	1129	33,497	–	–
DrugBank (ver5.1.7)	2616	782,405	–	–

### Feature preprocessing

We used compound structures and properties as features to train the feature generation model. All compounds were standardized using canonical SMILES. Then, the Morgan fingerprints and compound properties were retrieved using Mordred [33]. Mordred provides 1613 2-dimensional and 213 3-dimensional compound properties, and 771 properties were obtained after removing not-a-numbers. Then, to use the most significant features, we performed random-forest feature selection and selected the top-100 significant compound properties (see Additional file 2). Selected features were scaled between zero and one for each feature type because the range of each feature varied. The above process was completed for all compounds of LINCS and TWOSIDES, and the scaling range was based on LINCS compounds because the model was trained with it. In the case of external data identifiers (IDs) compound ID (CID) conversion from DrugBank ID to PubChem CID was done for DrugBank compounds to check duplicates between datasets. Only compounds having SMILES information were extracted, and corresponding interactions were filtered, including mirror pair removal.

### Feature generation model

The purpose of the feature generation model is to generate a vector of drug-induced gene expression given a compound. The model was trained with LINCS L1000 data. Compound structures were represented as fingerprints and properties were used as input features; each value passed a simple dense neural network. Then, both latent representations were concatenated to output 978-dimensional vectors. The model was trained and evaluated by the cross-validation method to train with as many data points as possible. Owing to the relatively small data size compared with the feature dimension, various techniques were used to handle overfitting, such as the L2 regularizer with batch normalization. Furthermore, the cosine annealing method, a learning-rate scheduling method that avoids the local minimum, was used. As a loss function, we used the mean-squared error, whose formula is: $\frac{1}{{\text{n}}}\mathop \sum \limits_{{{\text{i}} = 1}}^{{\text{n}}} \left( {{\text{y}}_{{{\text{pred}}}} - {\text{y}}_{{{\text{true}}}} } \right)^{2} ,$ where ${\text{y}}_{{{\text{pred}}}}$ and ${\text{y}}_{{{\text{true}}}}$ each indicates predicted and actual labels. The specification of the feature generation model can be found in Additional file 1: Table S3.

### DDI prediction model

The DDI prediction model takes inputs in the form of triplet (i.e., drug $i$, drug $j$, and side effect $r$). First, drug features are engineered through a gating mechanism to represent the co-administration effects of drug pairs. Drug feature formulation is the process of selecting important genes related to interactions between given drugs. Here, we applied gated linear units (GLUs) to adjust the information flow of each drug feature based on drug pairs [34]. GLU is a simple gating mechanism originally developed for language models to control information propagating when passing layers as output gates with long short-term memory.

1 $$GLU\left( {A,B} \right) = A \otimes \sigma \left( B \right).$$

Equation (1) takes two inputs, $A$ and $B$, where $A$ is the output of the former layer, which is the information to propagate, and the other $B$ is the input of the gate $\sigma$, which controls how much of $A$ to use by applying sigmoid non-linearity. With the element-wise multiplication of two parts, it works as an output gate. As shown in Fig. 1B, two hidden layers of *drug i* and *drug j* were concatenated to represent co-administration and the dense layer was sigmoid-activated to output the same dimension as previous layers. Then, the vector is multiplied to the previous layer. Through multiplication, the vector works as a weight vector on gene expression values. Finally, an additional dense layer was followed for dimension reduction. Here, two dense layers, the one before co-administration module and one for dimension reduction, were shared between the two drugs to handle non-directionality of pairs.

Next, engineered drug features are projected into the side-effect space via translating embedding. Here, we applied the concept of translating embeddings [35, 36]. The embedding space represents each side effect, and both head and tail nodes each of which represents the start and the end point connected by a relation vector are given the drugs. Hence, a drug pair may have positive or negative interactions for a given side effect. Therefore, we considered a side effect as a relationship between two drugs and applied the margin-based loss function, implying positive pairs of drug combinations are positioned closely on the given side-effect space [37]. To train the model, we calculated a score and used the margin-based loss as described in a previous paper [23]. The score of a drug pair, $d_{i}$ and $d_{j}$, given side-effect $r$, is calculated by Eq. (2).

2 $$S\left( {d_{i} ,d_{j} ,r} \right) = \left| {\left| {M_{rh} h + r - M_{rt} t} \right|} \right|_{2} + \left| {\left| {M_{rh} t + r - M_{rt} h} \right|} \right|_{2} ,$$

where $h$ and $t$ are embedding vectors of entities that are drug latent representations, $r$ is an embedding vector of a relation, and $M_{rh}$ and $M_{rt}$ are mapping matrices multiplied to $h$ and $t$ to project drug features to side effect spaces, respectively. Here, we considered either direction because drug combination data do not have directionality. Then, the L2 norm was applied to calculate the distances. The specification of the DDI prediction model can be found in Additional file 1: Table S4.

The final output is the score (distance) of a triplet. Therefore, to train the model, a margin-based loss was used. The loss is defined as

3 $$L = \mathop \sum \limits_{{\left( {r \in R_{se} } \right)}} {\text{max}}\left( {0,\mathop \sum \limits_{{\left( {d_{i} ,{ }d_{j} } \right)^{ + } \in D_{r} }} S\left( {\left( {d_{i} ,{ }d_{j} } \right)^{ + } ,r} \right) - \mathop \sum \limits_{{\left( {d_{i} ,{ }d_{j} } \right)^{ - } \in D_{r} }} S\left( {\left( {d_{i} ,{ }d_{j} } \right)^{ - } ,r} \right) + margin} \right),$$

where $R_{se}$ represents side effects, $D_{r}$ represents a set of drug pairs given a side effect, ${ }r$ containing both positive and negative pairs marked as ±. The classification model was trained to derive the scores that differ by the margin between positives and negatives.

### Experimental setup

To test the robustness of the model in a rigorous way, we prepared differently split datasets for each evaluation. The first test predicted unknown interactions between known drugs (i.e., “unseen” interactions). Here, a known drug is the one used to train the model. The second case predicted interactions between a known drug and an unknown drug, meaning that either drug is seen in the model by training. We refer to this case as “one-unseen” drug. The last case (i.e., “both-unseen” drug) predicted interactions between arbitrary drugs that were not used for training the model. The first case contains the objectives of previous studies; thus, we compared our model using this condition. However, the latter two cases were more challenging because the drugs were not learned by the model, resulting in insufficient information during the training phase.

We split the dataset based on each case and data type for training, validation, and testing. First, for the case of unseen interaction prediction, we split the total triplets into training and testing at a ratio of nine-to-one. Then, 1% of the training set was sampled for validation (see Additional file 1: Fig. S1). Moreover, negative triplets were obtained for each set by randomly sampling $d_{j}$ for each ($d_{i} , r$) as in a previous study [21]. The negatives were sampled so that the ratio of positives to negatives to be equal (see Additional file1: Table S7, Fig. S2, and Additional file 3). Triplets were selected to be exclusive to all sets. Moreover, the reversed pairs are considered for all datasets, because drug pairs do not have directionality—drug 1–drug 2 and drug 2–drug 1 should have the same side effects.

For evaluation, we compared the prediction performance with previous studies—one is the first paper for predicting polypharmacy side effects using graph convolutional networks, and the other utilized the L1000 data [21, 28]. Especially we implemented the ANN model to predict with our dataset because the proposed architecture can be applied for ‘one-unseen’ and ‘both-unseen’ cases.

The parameters of each model are listed in Additional file 1: Table S5, S6. In the feature generation model, we compared the performances of models with different lengths of fingerprints, and the best case was selected. The different embedding sizes in the DDI model were tested, but there were no significant differences in performance. Moreover, different sizes of input features were tested on DDI model to examine whether the size of gene expression features affects model performance. As shown in Additional file 1: Fig. S11, the original features performed the best despite its dimension and also allowed feature analysis.

### External validation process

We downloaded and preprocessed two versions of DrugBank (versions 5.0.0 and 5.1.7) for external validation. We first predicted potential DDIs using the old version (v. 5.0.0, released 2016) and evaluated the prediction performance against the new version (v. 5.1.7, released 2020) by checking if the predicted potential DDIs can be found in the new version. For this purpose, the 603,259 unknown drug pairs obtained to exclude positive pairs from all possible drug combinations of DrugBank 5.0.0 drugs were predicted. The input of our model was designed to intake a triplet of two drugs and their corresponding side effects, whereas the DrugBank provides DDI information with no explicit side-effect terms (see Additional file 1: Tables S1, S2). Therefore, to manage the knowledge discrepancy of the two databases, we constructed positive and negative DDI datasets for DrugBank using the following procedures. First, to construct the positive DDI dataset, we collected drug pairs that were reported as having side effects. Next, for the negative DDIs, the negative drug pairs were obtained by excluding positive drug pairs from all possible drug combinations. Then, to evaluate prediction results with a triplet form of representation from our model, we referred to the average number of side effects of the drug pair in TWOSIDES and used the average value as the cutoff for determining the DDI positive (see Additional file 1: Fig. S13). The average number of side effects for each drug pair was ~ 65; thus, hence pairs having more than 65 side effects were labeled “positive” and “negative” otherwise.

## Results and discussion

### Performance evaluation of gene-expression generation model

The feature generation model was trained using the entire LINCS L1000 dataset, and the performance was evaluated by how closely it predicted the actual expression values. We used the Pearson correlation coefficients between the golden standards and predicted expression values as an evaluation metric. The distribution of expression values can be found in Additional file 1: Fig. S3. The feature model was selected considering both metrics via tenfold cross-validation and further validated through external validation by splitting dataset into train-test sets. To achieve better performance, we trained for 1000 epochs and checked the loss and Pearson correlation coefficient (Additional file 1: Fig. S5, Table S8). The model achieved a correlation of 0.518 and a training loss of 1.834. The performance comparisons with models using different features are described in Additional file 1: Fig. S4. Moreover, the distributions of true expression values from LINCS and predicted values for TWOSIDES are compared as shown in Additional file 1: Fig. S6. The chosen model was then trained with the entire dataset to check all compounds because the dataset was relatively small.

### Performance evaluation of DDI prediction

**Fig. 2:**
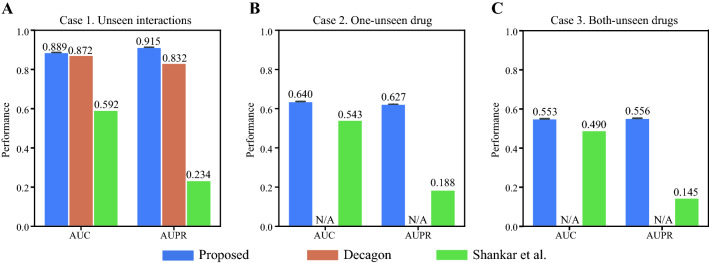
Model performances of each DDI prediction case. The model performances are the average values of all side effect results. We compared our models to Decagon (orange) and the ANN model; from Shankar et al. (green). **A** Our model showed good performances compared to previous studies. The performance of ANN model is implemented results with our dataset, while the reported performances in the original paper are AUC of 0.634, accuracy of 0.787, and F1 of 0.631 obtained by averaging prediction performances of 243 side effects. **B**, **C** Decagon was not applicable to predict unseen drugs, and the ANN model was implemented

The performance of the proposed model was evaluated under robust conditions. We used three test datasets that were exclusive to training: unseen interaction, one-unseen, both-unseen (see Materials and methods section for details). The first case is the general case of predicting new interactions between known drugs; this is the focus of most extant reports. The area-under-the-curve (AUC) and the area-under-the-precision-recall (AUPR) values were calculated using the prediction scores (see Additional file 1: Additional Materials and Methods). Sensitivity and specificity values of each side effect were calculated using the optimal threshold values (see the section “Experimental setup”). As shown in Fig. 2, the prediction model presented high performance with an AUC of 0.889 and an AUPR of 0.915 for unseen interactions (Fig. 2A). We noticed that the model showed varied performance for each side effect. The best and worst performing side effects are listed in Table 3.

**Table 3:** Best- and the worst-performing side effects

Best-performing side effects			Worst-performing side effects		
Side-effect Name	AUC	AUPR	Side-effect Name	AUC	AUPR
Hypermetropia	0.963	0.967	Brain abscess	0.803	0.848
Epicondylitis	0.954	0.962	Renal colic	0.814	0.853
Pneumoconiosis	0.953	0.961	Impetigo	0.817	0.856
Fibrosing alveolitis	0.950	0.966	Cerebral thrombosis	0.832	0.857
Epididymitis	0.946	0.954	Ejaculation premature	0.807	0.858

In addition, we validated the DDI prediction model to confirm robustness. First, we investigated whether the model performance is related to the frequency of side effects or similarities between side effects. There were no significant correlations between performances and the frequency of side effects. Also, the model showed consistent performance regardless of side effect similarities and categories in terms of concept level (see Additional file 1: Figs. S7, S8). Next, the triplets include cases where the side effect of one drug is equal to the polypharmacy side effect (see Additional file 1: Fig. S9). However, it is hard to determine if a reported polypharmacy side effect is induced by either drug because the dataset is originally from FAERS. Therefore, we compared the predicted DDI scores between original pairs and either one drug in the pair, assuming that the model may output similar scores if polypharmacy side effects are highly correlated to single side effects. The pairs of the one-sided drugs resulted in different predicted scores while the original and reversed pairs showed similar scores resulting in the Pearson correlation coefficient of 0.952 and R^2^ of 0.905 (see Additional file 1: Fig. S10).

Moreover, as the Decagon reported their best and worst lists, we compared our results similarly. Our model showed better AUPR in four out of the ten best-performing side effects and outperformed all worst-performing side effects by up to 23% (see Additional file 1: Table S10). To confirm the predictive power of the model, we extracted the prediction result of previously known fatal drug interactions. Warfarin, an anticoagulant, is known to have dangerous interactions with non-steroidal anti-inflammatory drugs (NSAIDs) resulting in serious bleeding, especially gastrointestinal (GI) bleeding. Also, amiodarone, which is one of the antiarrhythmic medications, is known to induce muscle-related pain when combined with statins. In the test set of unseen interactions, we found those drug pairs with several side effects. Our model correctly predicted those side effects and there are corresponding types as listed in Table 4.

**Table 4:** The list of previously known drug pairs and positively predicted side effects

Drug 1	Drug 2	Predicted known side effects
Warfarin	Naproxen	Bleeding gums, coughing blood, blood disorder, Epistaxis
	Ibuprofen	Abdominal pain
	Indomethacin	Lower GI bleeding, subarachnoid haemorrhage
	Meloxicam	Haematochezia, malnourished
Amiodarone	Loavastatin	Arterial pressure NOS decreased, cramp
	Rosuvastatin	Asystole
	Fluvastatin	Rhabdomyolysis

Next, for the “one-unseen” prediction problem, our model achieved an AUC of 0.640 and an AUPR of 0.627. Unlike the unseen interaction case, the Decagon model cannot be applied to “one-unseen” drug prediction because it uses a graph structure when representing interactions where nodes are fixed. In the case of the ANN model (Shankar et al.), it showed low performances, especially regarding AUPR (Fig. 2B). For the “both-unseen” case, the model showed limited performance. The average performances had an AUC of 0.553 and an AUPR of 0.556.

In all cases, our model performed better than previous models. Decagon shows a good performance in predicting unseen interactions, but it is not applicable to predict interactions with unseen drugs because unseen drugs are not connected to the network [21]. Meanwhile, the ANN model worked by Shankar et al. could be implemented for case 2 and 3 [38]. However, it showed relatively poor performance with our dataset. The performance drop might be the result of predicting more types of side effects while the original study focused on only 243 side effects. Furthermore, the ANN model concatenates all features resulting in high dimension features, and it may be challenging for a simple neural network to capture interactions with unseen drugs. The prediction performances for each side effect type are listed in Additional files 4, 5, and 6 for each case with detailed metrics including sensitivity, specificity and precision.

**Fig. 3:**
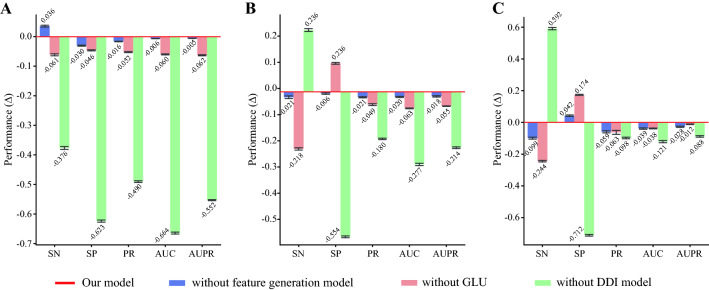
Ablation study results on each DDI case. The differences in performance for each case—unseen interactions (**A**), one-unseen drug (**B**), and both unseen drugs (**C**). In all cases, the model without DDI prediction model (green) showed drastic decrease in performance which indicates that it contributed the most

We also investigated which model contributed the most to performance improvement. The models without each feature generation model and DDI model were tested. Also, the model without the co-administration module of the GLU was compared. As shown in Fig. 3, the performance drastically dropped when DDI model with the GLU and the score calculation module was substituted with simple neural networks. Next, the performance slightly decreased without the GLU module while the model without the feature generation model showed little change in performance. This result indicates that both the co-administration module and the embedding contributed the most to the model performance.

### External validation using DrugBank dataset

**Fig. 4:**
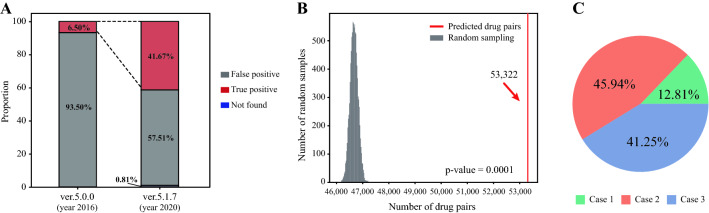
External validation with DrugBank datasets. **A** Change in the proportions of true positives and false positives predicted in DrugBank verv5.0.0 compared with v.5.1.7. More than 35% of drug pairs were found to be interactions present in the new version. **B** Result of the permutation test. The grey distribution is from 10,000 random samplings, and the red line indicates the number of drug pairs found in the updated DrugBank version. **C** Proportion of each DDI case of validated drug pairs. Most cases were one-unseen and both-unseen drug cases

We next performed external validation using the DrugBank datasets. The validation process is described in Materials and methods section. We made predictions for all possible pairs from DrugBank version 5.0.0, including 33,497 positive and 603,259 negative pairs from 1129 drugs. With the threshold of 65 obtained from the trained model, 9543 true positives and 137,201 false positives were detected. To validate the prediction performance of the model, we checked predicted DDIs in the earlier version (5.0.0) against the later version (5.1.7) to check whether predicted DDIs were found to be real DDIs in the future. Among 137,201 drug pairs, 53,322 drug pairs were detected in DrugBank v.5.1.7. As shown in Fig. 4A, in the old version, only 6.50% were true positives; however, the proportion increased by 41.67% in the updated version. Moreover, the permutation test was performed to confirm that the number of found DDIs was significant. We randomly sampled 53,322 drug pairs 10,000 times and confirmed how many were found in the new version. Figure 4B shows the distribution of the number of confirmed DDIs of random sampling, and the red line indicates the number of confirmed DDIs of the predicted result with a p-value of 0.0001. The validated drug pairs consist of all three cases—most were one-unseen and both-unseen drug cases—confirming that our DDIs prediction model has a high potential to detect unknown DDIs among drugs (see Fig. 4C and Additional file 1: Fig. S14 for details).

### Prediction of potential drug combinations

We further investigated the potential novel DDIs, which were not reported in the TWOSIDES database. Among the positively predicted drug pairs with ten different models, we confirmed 2733 pairs out of 19,811 annotated as DDIs in the DrugBank database on an average. (see Additional file 1: Fig. S12). The part of validated interactions is listed in Additional file 1: Table S9. With a stricter criterion of positively predicted from all models, we found 1072 potential DDIs that were not reported previously. (see Additional file 7) From this result, we can conclude that our proposed model shows robust prediction performance and can suggest potential DDIs.

### Exploration on drug features

**Fig. 5:**
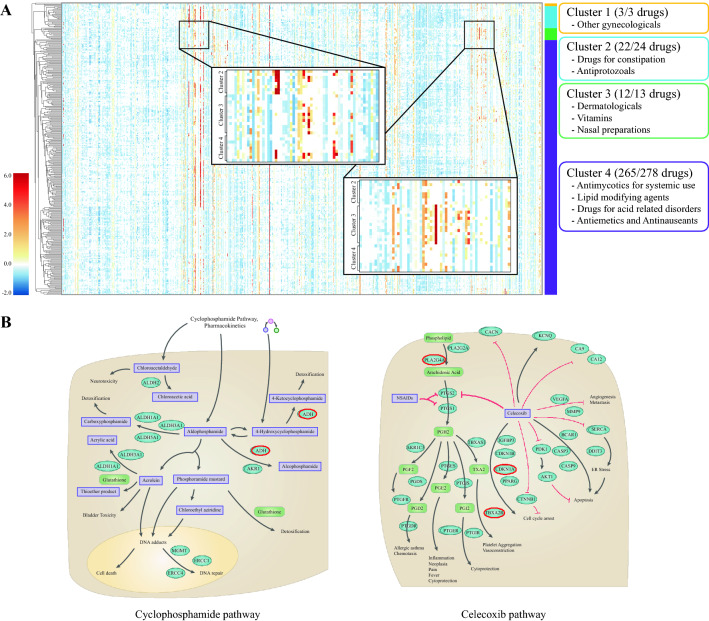
Analysis of drug dynamic features.** A** Visualization of changes in compound treated gene expressions depending on co-administered drugs. The heatmap shows the changed features of one drug affected by each paired drug. Hierarchical clustering was done considering the correlation of features, and drug categories of each cluster were labeled. **B** Pathway information of cyclophosphamide and celecoxib. Among highly attended genes, ADH5 in chyclophosphamide pathway and PLA2G4A, TBXA2R, and CDKN1A genes in celecoxib pathway were detected as highlighted in red

One of the main contributions of this study is the engineering of dynamic drug features that represent drug co-administration effects. This concept was implemented by a gating mechanism that differentiates latent representations of a drug when it was co-administrated with the other. Here, we observed the changes of latent drug representations of cyclophosphamide, which is one of the anticancer drugs. There were 318 drug pairs that were reported to have side effects when taken together with cyclophosphamide. We examined the changed features of cyclophosphamide in combinations with other drugs. As shown in Fig. 5A and Additional file 1: Fig. S15, we confirm that the latent representation of the cyclophosphamide varied with the paired drugs. Representation of each gene for DDIs were multiplied by the existing latent features according to their importance to generate different latent representations.

The following result shows that the model can capture the response of a same drug differently depending on the drug with which it is combined. Additionally, we analyzed which drugs had similar effects by clustering the latent representation values. Moreover, we checked whether the drugs of each cluster had any characteristics by examining the drug categories that are defined in DrugBank. We confirmed the Anatomical Therapeutic Chemical (ATC) codes of the drugs for each cluster and selected the code that appeared most through the enrichment test [39]. The therapeutic classes are not all exclusive, but representative classes are illustrated. Because the cluster 1, 2, and 3 consist of small number of drugs, there exist overlapping ATC codes. The most enriched codes in cluster 1 and 2 are ‘other gyncologicals’ and ‘drugs for constipation’. In the case of cluster 3, ‘dermatologicals’, ‘vitamins’, and ‘nasal preparations’ are unique codes, one of each was included. Among them, ‘nasal preparations’ is unique in all drug pairs with cyclophosphamide. The major cluster included various exclusive classes such as ‘antimycotics for systemic use’ and ‘lipid modifying agents’. The total list of ATC codes are listed in Additional file 8. Also, different side effect occurrences were detected for each cluster as listed in Additional file 1: Table S11. Finally, analysis on important genes was performed based on the attention values of each gene feature calculated via a GLU. Assuming that the genes receiving high attention values would be important for a given DDI, we analyzed highly attended genes in the interaction between cyclophosphamide and celecoxib using the pathway information obtained from the PharmGKB database [40, 41]. Based on each vector calculated from the two-drug information, 100 genes having the highest values were extracted. Then, we confirmed whether any genes corresponded to pathway-related genes. Hence, the ADH5 in the cyclophosphamide pathway and the PLA2G4A, CDKN1A, and TBXA2R in the celecoxib pathway were found among the top-100 genes (Fig. 5B). This result indicates that the GLU can capture molecular information from drug-induced gene expressions.

## Conclusion

In this study, we proposed a robust deep-learning model for predicting polypharmacy side effects. The main contribution of our work includes the construction of a gene-expression feature generation model that produces drug-treated gene expression signatures, the application of a gating mechanism to represent the co-administration effect of a drug pair, and a report of potential DDIs among drug pairs.

First, our model utilized gene-expression data to predict DDIs. Differing from previous studies that simply used structures or properties of drugs, our model used a gene-expression signal that represents the holistic view of the drug-induced effects of cells. However, because not all compounds had compound-treated gene-expression signatures, our model included a pre-trained feature generation model to produce compound features. We used compound structure and property information to generate expected compound-treated gene expressions. Then, the DDI prediction model processed expression features to output defined side-effect scores. The model was evaluated under three cases: predicting unseen interactions, one-unseen drug, and both-unseen drugs. For each case, data were split into training and testing sets for strict evaluation. The model showed competitive performance compared with previous studies of predicting unseen interactions.

Second, to mimic the effect of co-administration, we applied feature processing with respect to a gating mechanism, and the KG embedding algorithm was applied to handle multiple side effects per drug pair. Through the co-administration module, we aimed to reflect the changes in features by the other drug. Additionally, the translating embedding module resulted in a dramatic increase in performance.

Third, we reported potential DDIs that were not reported before. Based on the external validation using the DrugBank database, we can expect that the list of potential DDIs will help prevent harmful effects from co-administration. Furthermore, because the model can predict interactions with unseen drugs, it can possibly pre-detect unwelcomed reactions among compounds during the early drug-discovery process.

There still exist issues to be improved in the future. First, the datasets in this case were very sparse in terms of side-effect type. The small proportion of interactions between drugs were known, but the numbers were extremely sparse and imbalanced when considered with relation type. For this reason, rare side effects were excluded for the current model. This can be overcome with a combination of disease-related information, including pathways. Second, the identification of the side-effect mechanism remains challenging. Although our model utilized gene expression data, only a portion of human genes was selected, and the heterogeneity of cell lines induced noise. We constructed the feature generation model to supplement lack of experimental data, however, predicting transcriptiome-level features from drug structures and properties is both challenging and limited. With enriched drug response and network information signaling, all underlying mechanisms may soon be uncovered.

## Supplementary Information

**Additional file 1. **Additional information. Additional description on Materials and Methods, Results with Figures and Tables.

**Additional file 2. **LINCS compound properties. Total list of compound properties used for the feature generation model and each value.

**Additional file 3. **The number of positive and negative triplets for each side effect type. Detailed number of triplets for each side effect type in Case 1 dataset.

**Additional file 4. **DDI prediction model performance for Case 1. Side effect specific performance of DDI model in Case 1 (unseen interactions).

**Additional file 5. **DDI prediction model performance for Case 2. Side effect specific performance of DDI model in Case 2 (one-unseen drug).

**Additional file 6. **DDI prediction model performance for Case 3. Side effect specific performance of DDI model in Case 3 (both unseen drugs).

**Additional file 7. **List of predicted DDIs. The list of positively predicted triplets in unseen interactions test set. The validation labels from DrugBank are annotated.

**Additional file 8. **The list of ATC codes of drugs in each cluster from dynamic drug features of cyclophosphamide. The second level of ATC codes was obtained which represents therapeutic subgroups. The list is sorted by the odds ratio of the number of drugs in the cluster over the total number of drugs with the code.

## Data Availability

All codes are available at https://github.com/GIST-CSBL/DeSIDE-DDI

## Abbreviations

DDI: Drug-drug interaction
LINCS: Library of integrated network-based cellular signatures
ANN: Artificial neural network
SS: Signature strength
SMILES: Simplified molecular-input line-entry system
GLU: Gated linear unit
AUC: Area-under-the-curve
AUPR: Area-under-the-precision-recall
NSAIDs: Non-steroidal anti-inflammatory drugs
ATC: Anatomical therapeutic chemical
