# Supplementary material for: DeSIDE-DDI: interpretable prediction of drug-drug interactions using drug-induced gene expressions
Source: J Cheminform. 2022 Mar 4;14:9. doi: 10.1186/s13321-022-00589-5 (PMC8895921; doi:10.1186/s13321-022-00589-5)
Supplement: Supplementary file 1 — Additional file 1. Additional information. Additional description on Materials and Methods, Results with Figures and Tables. [file 13321_2022_589_MOESM1_ESM.docx]

Additional Information

DeSIDE-DDI: Interpretable prediction of drug-drug interactions using drug-induced gene expressions

*Eunyoung Kim^1^, Hojung Nam^§^*

School of Electrical Engineering and Computer Science, Gwangju Institute of Science and Technology (GIST), Buk-gu, Gwangju, 61005, Republic of Korea

Table of Contents

[**1.** **Additional Materials and Methods** 3](#_Toc91492441)

[**1.1.** **DrugBank data processing** 3](#_Toc91492442)

[**1.2.** **Feature information** 4](#_Toc91492443)

[**1.3.** **Model architecture** 4](#_Toc91492444)

[1.3.1. Feature generation model 5](#_Toc91492445)

[1.3.2. DDI prediction model 5](#_Toc91492446)

[1.3.3. Model parameters 6](#_Toc91492447)

[**1.4.** **Dataset split for model training, validation, and testing** 7](#_Toc91492448)

[**1.5.** **Validation process with DrugBank dataset** 10](#_Toc91492449)

[1.5.1. Validation of newly predicted interactions in the testing set 10](#_Toc91492450)

[1.5.2. External validation to confirm model robustness 11](#_Toc91492451)

[**1.6.** **Performance metrics** 11](#_Toc91492452)

[**2.** **Additional Results** 13](#_Toc91492453)

[**2.1.** **Performance of feature generation model** 13](#_Toc91492454)

[**2.2.** **Performance of DDI prediction model** 16](#_Toc91492455)

[2.2.1. Robustness of DDI prediction model 16](#_Toc91492456)

[2.2.2. Impact of weighted features in DDI prediction 20](#_Toc91492457)

[**2.3.** **Validation of predicted potential interactions in TWOSIDES** 22](#_Toc91492458)

[**2.4.** **Analysis on internal validation** 23](#_Toc91492459)

[**2.5.** **External validation** 24](#_Toc91492460)

[**2.6.** **Feature analysis** 26](#_Toc91492461)

#

# **Additional Materials and Methods**

## **DrugBank data processing**

DDI information was extracted from two databases: TWOSIDES and DrugBank. Instead of using original TWOSIDES data, we downloaded the BioSNAP dataset, which provides refined DDI information excluding additional statistics. In the case of DrugBank, we extracted DDIs from the raw data of two versions (v. 5.0.0 and 5.1.7). Unlike TWOSIDES data, DrugBank describes DDIs with sentence representations instead of explicit side-effect terminologies. Example data are listed in **Tables S1, S2**. To manage the knowledge discrepancy of the two databases, we selectively took the information of drug pairs from DrugBank DDIs. Then, we constructed positive and negative DDI datasets for DrugBank using the following procedure. First, to construct the positive DDI dataset, we collected drug pairs that were reported as having side effects. Next, for the negative DDIs, the negative drug pairs were obtained by excluding positive drug pairs from all possible combinations. Then, to evaluate prediction results that have triplet forms of representation from our model, we referred to the average number of side effects of the drug pair in TWOSIDES and used the average value as the cutoff for the determination of the DDI positive. A more detailed process is described in the Validation Process with DrugBank Dataset chapter in **Additional Materials and Methods**)**.**

**Table S1.** Example of DDI information representation in the BioSNAP dataset

| **STITCH 1** | **STITCH 2** | **Polypharmacy Side Effect** | **Side-effect Name** |
| --- | --- | --- | --- |
| CID000002173 | CID000003345 | C0151714 | hypermagnesemia |
| CID000002173 | CID000003345 | C0035344 | retinopathy of prematurity |
| CID000002173 | CID000003345 | C0004144 | atelectasis |
| CID000002173 | CID000003345 | C0002063 | alkalosis |

**Table S2.** Example of processed DrugBank DDI information (v. 5.1.7)

| **Drug1 ID** | **Drug1** | **Drug2 ID** | **Drug2** | **Description** |
| --- | --- | --- | --- | --- |
| DB00001 | Lepirudin | DB06605 | Apixaban | Apixaban may increase the anticoagulant activities of Lepirudin |
| DB00001 | Lepirudin | DB06695 | Dabigatran etexilate | Dabigatran etexilate may increase the anticoagulant activities of Lepirudin. |
| DB00001 | Lepirudin | DB01254 | Dasatinib | The risk or severity of bleeding and hemorrhage can be increased when Dasatinib is combined with Lepirudin. |
| DB00001 | Lepirudin | DB01609 | Deferasirox | The risk or severity of gastrointestinal bleeding can be increased when Lepirudin is combined with Deferasirox. |

## **Feature information**

The features include compound structures and properties. We used the Morgan fingerprints as structure information. The parameters used include a radius of two and output vector lengths of 1,024 bits. Additionally, compound properties were obtained using Mordred. The total number of properties provided by Mordred is 1,826, including 1,613 2-dimensional and 213 3-dimensional properties, but not all can be calculated for all compounds. After removing unobtainable properties, 771 remained. Then, we constructed a random forest model to select important features. Using the scikit-learn Python library, we trained a model b varying the *max_depth* parameter to derive feature importance. All features were ranked based on their importance, and the top-100 were selected. Then, the selected features were normalized to range from zero to one because the ranges of each feature type varied. The selected features and corresponding values can be found in **Additional File 2**.

## **Model architecture**

Our model consists of two sub-models. The first is a feature generation model that was pre-trained with LINCS L1000 compounds and paired compound-treated gene expressions. This was used to calculate input features of the DDI prediction model. The specifics of each model are described.

### Feature generation model

The feature generation consists of simple neural networks. Each compound’s fingerprints and properties pass through separate dense layers and are concatenated to output predicted gene expressions. The architecture is described in **Table S3**.

**Table S3.** Specification of the feature generation model

| **Layer info** | **Output shape** | **Connected to** | **Details** |
| --- | --- | --- | --- |
| FP_input | 1,024 |  | Morgan fingerprints |
| Property_input | 100 |  | Compound properties |
| FP_dense1 | 512 | FP_input | Activation: rectified linear unit (ReLU) |
| FP_dense2 | 128 | FP_dense1 | Activation: ReLU |
| Property_dense | 128 | Property_input | Activation: ReLU |
| Concatenate | 256 | [FP_dense2, Property_dense] |  |
| Output | 978 | Concatenate | Activation: tanh |

### DDI prediction model

The predicted drug-treated gene expressions of two drugs in a drug pair were used as inputs to the DDI prediction model. With an additional GLU for information control, the drug layers consisted of dense layers. Then, latent representations of drugs were projected to each side-effect space to calculate side-effect scores. Both directions were considered and summed to output the final scores. The details of architecture can be found in **Table S4**.

**Table S4.** Specification of the DDI prediction model

| **Layer info** | **Output shape** | **Connected to** | **Details** |
| --- | --- | --- | --- |
| Drug1_input | 978 |  | Drug1 input |
| Drug2_input | 978 |  | Drug2 input |
| Drug1_dense1 | 978 | Drug1_input | Activation: linear |
| Drug2_dense1 | 978 | Drug2_input | Activation: linear |
| Concatenate | 1,956 | [Drug1_dense1, Drug2_dense1] |  |
| GLU_drug1_dense | 978 | Concatenate | Activation: sigmoid |
| GLU_drug2_dense | 978 | Concatenate | Activation: sigmoid |
| GLU_drug1_multiply | 978 | [Drug1_dense1, GLU_drug1_dense] | Element-wise multiplication |
| GLU_drug2_multiply | 978 | [Drug2_dense1, GLU_drug2_dense] | Element-wise multiplication |
| Drug1_dense2 | 100 | GLU_drug1_multiply | Drug1 embedding |
| Drug2_dense2 | 100 | GLU_drug2_multiply | Drug2 embedding |
| SE_input | 1 |  | Side effect input |
| SE_embedding | 100 | SE_input | Side effect embedding |
| SE_embed_head | [100,100] | SE_input | Mapping matrices |
| SE_embed_tail | [100,100] | SE_input |  |
| Projection_dot_Mh_dx | 100 | [SE_embed_head, Drug1_dense2] | $M_{rh}h$ |
| Projection_dot_Mt_dy | 100 | [SE_embed_tail, Drug2_dense2] | $M_{rt}t$ |
| Projection_dot_Mh_dy | 100 | [SE_embed_head, Drug2_dense2] | $M_{rh}t$ |
| Projection_dot_Mt_dx | 100 | [SE_embed_tail, Drug1_dense2] | $M_{rt}h$ |
| Score1_Add | 100 | [Projection_dot_Mh_dx, SE_embedding] | Calculation of distance (1) |
| Score1_Subtract | 100 | [Score1_Add, Projection_dot_Mt_dy] |  |
| Lambda1_L2Norm | 1 | [Score1_Subtract] |  |
| Score2_Add | 100 | [Projection_dot_Mh_dy, SE_embedding] | Calculation of distance (2) |
| Score2_Subtract | 100 | [Score2_Add, Projection_dot_Mt_dx] |  |
| Lambda2_L2Norm | 1 | [Score2_Subtract] |  |
| Output | 1 | [Lambda1_L2Norm, Lambda2_L2Norm] | Addition of two scores |

### Model parameters

Given model architectures, there are several hyperparameters to be chosen. The Table S5 and S6 represent the parameters used for each model. For the feature generation model, the different lengths of fingerprints were tested. On the other hand, the different embedding sizes of drugs and side effects in the DDI prediction model were tested. Here, the larger embedding size caused the larger model size resulting in high memory usage and longer time to train. For both models, Adam optimizer was used as an optimizer.

**Table S5.** Parameter settings of the feature generation model

| **Parameters** | **Values** |
| --- | --- |
| Learning rate | 0.0001 |
| Batch size | 64 |
| ECFP length | [512, 1,024, 2,048] |

**Table S6.** Parameter settings of the DDI prediction model

| **Parameters** | **Values** |
| --- | --- |
| Learning rate | 0.0001 |
| Batch size | 1,024 |
| Embedding size | [10, 50, 100, 150, 200] |

## **Dataset split for model training, validation, and testing**

There are three cases of interactions that can occur in the real world: predicting unknown interactions between known drugs, between known and unknown drugs, and between unknown drugs. These cases are named “unseen interactions”, “one-unseen drug”, and “both-unseen drugs” respectively. Here, “unseen” indicates that those are not used to train the model, as shown in **Figure S1.A.** Depending on which is blinded to the model, we split the dataset into training, validation, and testing sets differently.


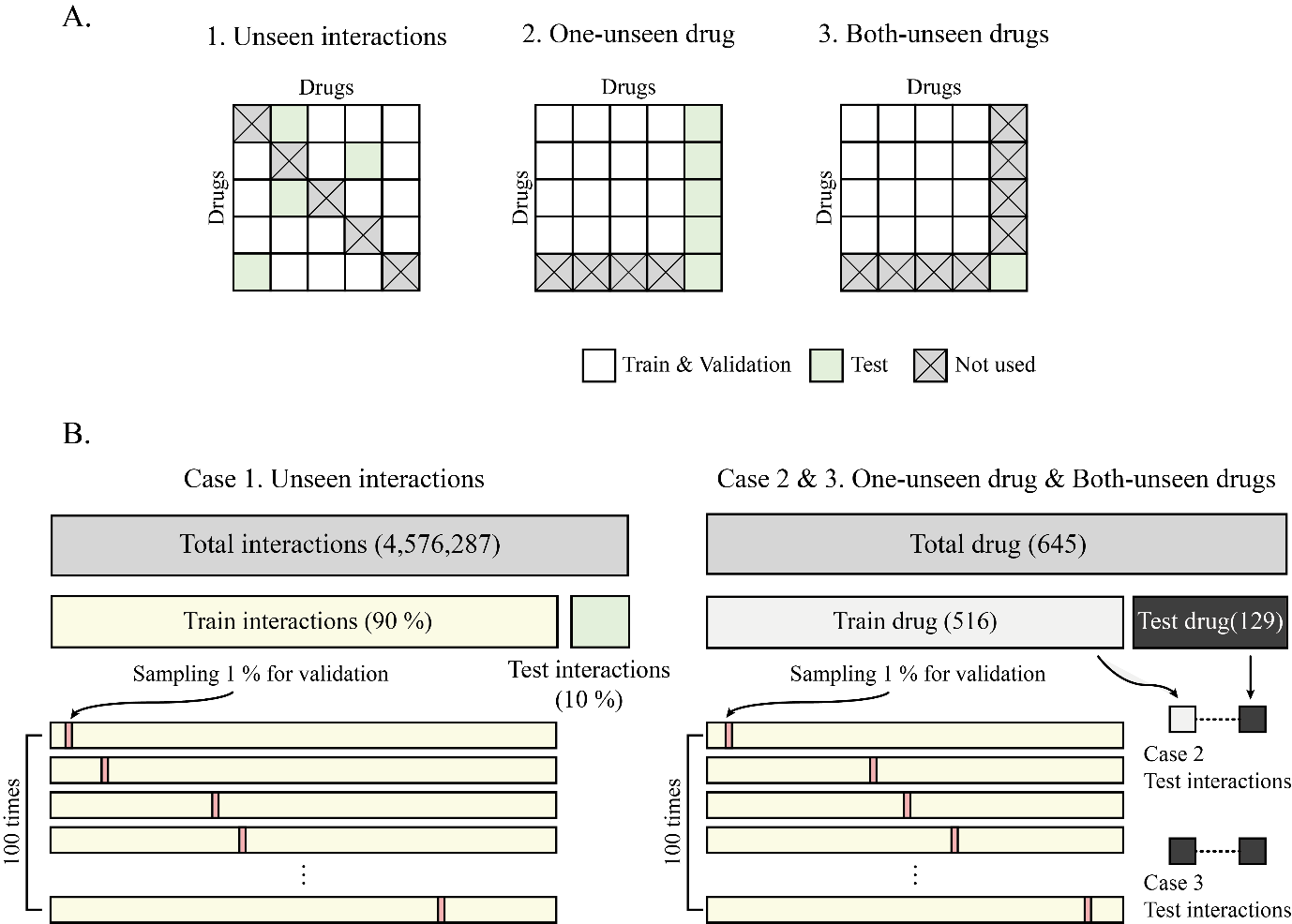


**Figure S1.** A. Illustration of data split for each case. Each cell represents the interactions between drugs. Depending on each case, intended interactions to predict are different. B. Illustration of the data split process of each prediction case.

To construct the prediction model for unseen interactions, we split total triplets in terms of interactions into training and testing sets (**Figure S1.B**). Then, 1 % of all train triplets was randomly sampled for validation. Notably, each side-effect frequency differed. Although several side effects were frequent, meaning that it accounted for a large proportion of the total data, some were relatively infrequent. Therefore, during sampling, the size of each side-effect must be considered to avoid sampling certain parts too many times and to obtain thresholds of all types (**Figure S2**). The validation set was used to find the optimal score threshold that chooses the value to label positive or negative. Because the size of the training dataset was large, a sampling approach was used and repeated 100 times. The threshold was determined when both sensitivity and specificity were at their highest. Additionally, the optimal score threshold was calculated for each side-effect type. On the other hand, to predict “one-unseen” and “both-unseen” drugs cases, we split the drugs beforehand to blind the information. Total drugs were split into training and testing drug sets. Here, we found duplicated drugs between LINCS and TWOSIDES; therefore, those were included in the training set. The same sampling strategy was applied on the training set as the unseen interaction prediction case. In all prediction tasks, models were trained with train triplets and, side-effect score thresholds were calculated using the validation sets. Finally, the model was evaluated with testing sets to obtain prediction performance. Here, the case 2 testing sets consist of interactions between training and testing drug sets, whereas case 3 includes interactions between drugs in the testing drug set. The number of triplets used for each case is listed in **Table S7**. Also, the detailed number of triplets of each side effect type for case 1 is listed in **Additional File 3**.

**Table S7.** Size of training and testing sets used in each case. For each case of triplets, upper cell represents the number of positive triplets and the lower represents the number of sampled negatives.

|  | **Case 1**  **(Unseen interaction)** | | **Case 2**  **(One-unseen drug)** | | **Case 3**  **(Both-unseen drugs)** |
| --- | --- | --- | --- | --- | --- |
|  | # Drugs | # Triplets | # Drugs | # Triplets | |
| Training | 645 | 4,118,706 | 516 | 3,040,662 | |
|  |  | 4,118,259 |  | 3,040,396 | |
| Testing | 645 | 457,581 | 129 | 1,384,866 | 150,759 |
|  |  | 457,579 |  | 1,384,866 | 150,707 |


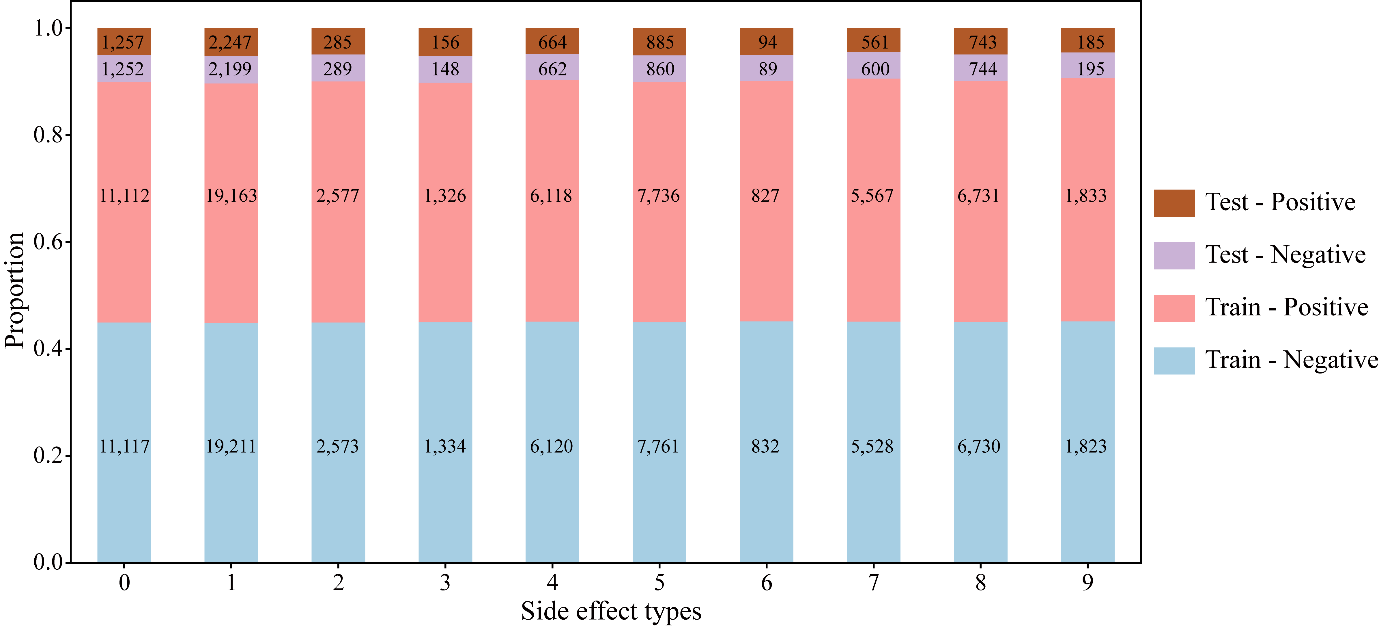


**Figure S2.** The number of drug pairs and proportion of each sets per side effet types. As the number of drug pairs for each side effect – frequency – differs, we split trainining and testing set into the same ratio for all side effects. Then, negative sampling was performed as its size to be as same as to the corresponding positive sizes. Each number represents the number of triplets in each set.

## **Validation process with DrugBank dataset**

An external validation was done with the DrugBank dataset. Owing to the difference in DDI description level, another approach was needed for validation. The classification model took inputs in the pre-defined triplet form; therefore, a single triplet of a drug pair and one side effect cannot represent the existence of interactions at the molecular level. To handle this problem, an additional approach was needed.

### Validation of newly predicted interactions in the testing set

We regard the predicted false positives out of the total test set as the newly predicted positives. Because these were not negatives but were instead unknown interactions, the DDI information in DrugBank was used to verify the predictions as possible interactions that were predicted to be close to positive. It is straightforward to match the predicted triplet to DrugBank. By ignoring the side-effect part of the triplets, only the drug-pair information can be retrieved and checked to see if it is found in DrugBank.

### External validation to confirm model robustness

When external validation was completed, the DrugBank dataset was utilized in a different way. In this case, we tested the previous version against the latest version of DrugBank. When we predicted the previous version, we confirmed that the predicted false positives were found in the latest version. To obtain false positives, labeling criteria were needed to determine which drug pairs were positive or negative in terms of the TWOSIDES side effects. We assumed that if a drug pair has more side effects than the average number in the positive training set, the drug pair will have interactions. To confirm the average numbers of side effects, 180,666 unique pairs were selected out of a total of 8,236,965 triplets, and the numbers of side effects were measured. Among those, the values of the pairs having side effects apart from the pair having zeros for all side effects (i.e., the pair that was all negative) were analyzed.

## **Performance metrics**

In this study, we evaluated two prediction models with different performance metrics as their tasks are different. For the feature generation model, we evaluated the model with the mean squared error (MSE) and Pearson correlation coefficient each of which measures how predicted values are far apart and how predicted expression values follow the tendency of true values.

The DDI prediction model was evaluated using five different metrics – sensitivity, specificity, precision, the area-under-the-curve (AUC), and the area-under-the-precision-recall (AUPR):

$$Sensitivity=\frac{\mathrm{TP}}{TP+FN}$$

$$Specificity=\frac{\mathrm{TN}}{TN+FP}$$

$$Precision=\frac{\mathrm{TP}}{TP+FP}$$

,where TP is true positive, TN is true negative, FP is false positive, and FN is false negative. The AUC is the area under the receiver operating characteristic (ROC) curve which plotted by true positive rate and false positive rate. The AUPR is the area under the curve of true positive rate and precision. The performance was calculated for each side effect type with the defined threshold.

# **Additional Results**

## **Performance of feature generation model**

The goal of feature generation is to understand how well a model can generate expected gene expression signatures when a compound is treated. Therefore, we fit the model with the given training dataset. The model was trained using the whole dataset and validated by predicting the dataset to see how well the model can reproduce ground truth. This is a challenging task because compound reactions undergo complex biological processes. The model was evaluated by mean-squared error and correlation coefficient. Each metric indicates how a model closely predicts the value and how much the model can predict the tendency. The range of expression values is -10 to 10, and the standard deviation was 1.6, indicating that most of values were close to zero, as shown in **Figure S3**. This data distribution can lead to low correlations with only the error-loss function when the predicted value has the opposite sign, whereas the real value is small. We attempted different options of Morgan fingerprints in terms of radius and output length; the use of 1,024 bits calculated with the radius of two resulted in the best performance.


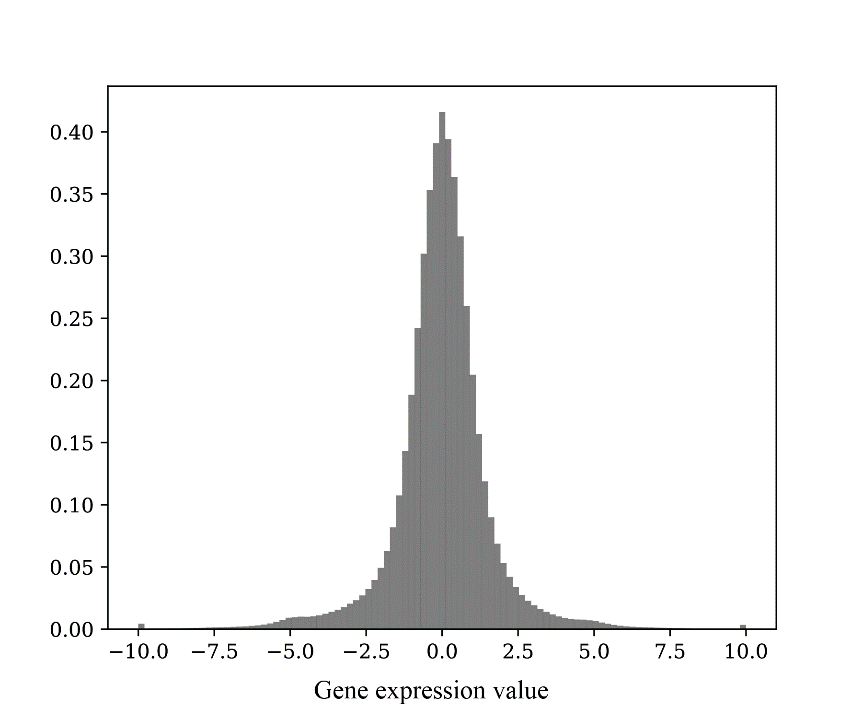


**Figure S3.** Distribution of LINCS L1000 compound-treated gene expression values

For the feature generation model, 10-fold cross-validation was used. When selecting the feature generation model, we compared it to others using different types of feature information: structure-only, property-only, and features without min-max scaling. Apart from the property-only model, all three models showed similar performance (**Figure S4**). However, we selected the model having both structure and property features because most compounds showed high structural similarities. The structure-only model tended to output similar expression values with structurally similar compounds. This can induce similar expression values for all compounds because most showed structural similarities higher than 0.8. To train the model to make distinctions regardless of compound structures, we utilized compound properties that can have different values with similar structures. Moreover, we scaled the values to between zero to one because fingerprints are binary values and properties vary to include the number of specific atoms or functional groups that depend on the size of the molecule. After selecting the model, we trained it using the entire dataset to train for all possible compounds to overcome the small number of data and the performance of the following model is shown in **Figure S5**. The model was also validated in an external validation manner by splitting the dataset into training and testing sets and evaluating with the testing set. We performed a random split 10 times and reported the average performance (**Table S8**). Here, we additionally validated prediction results with the coefficient of determination (R^2^) and the concordance index (C-index). The R2 was calculated as $R^{2}=1-\frac{RSS}{TSS}$, where the RSS is the residual sum of squares and the TSS is the total sum of squares. The low R^2^ resulted from the high residual sum of squares mainly from the difference between predicted and real values in the tails of the distribution. Therefore, the C-index which measures the order of the predictions are further calculated. After validation, the final model was retrained with the entire dataset.

Additionally, to confirm how well the feature generation model produced features, we compared the expression values of each dataset. **Figure S6** shows the feature value distributions. The blue color represents the real expression values from LINCS-only compounds. Orange represents the predicted expression values of compounds in only the TWOSIDES dataset, and the green represents the values of 161 both common compounds. The number of each compound set varies, and predicted values had less variation, showing that it was difficult for the model to predict tail-sided values.


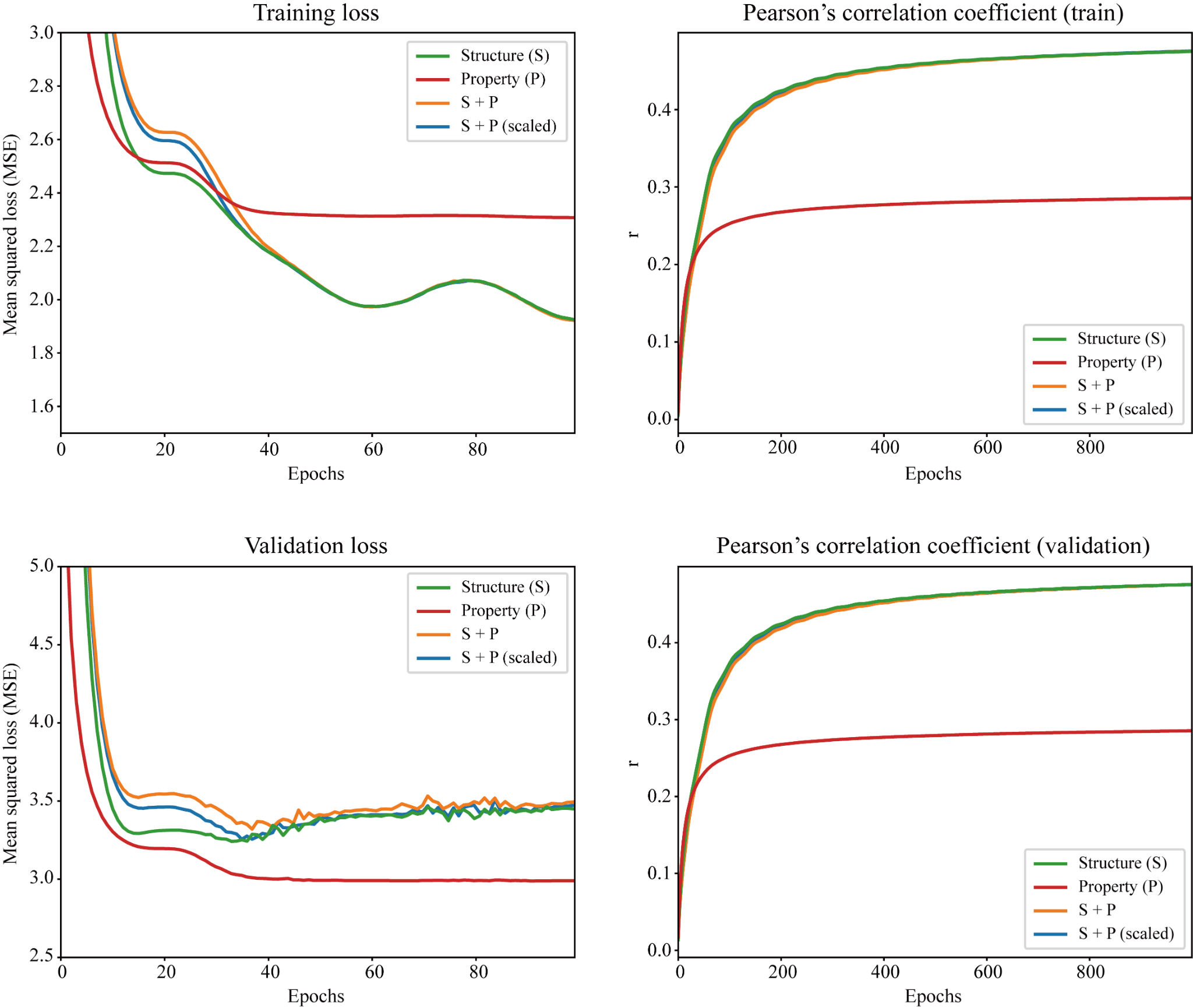


**Figure S4.** Comparison of cross-validation through loss and coefficient changes along epochs.


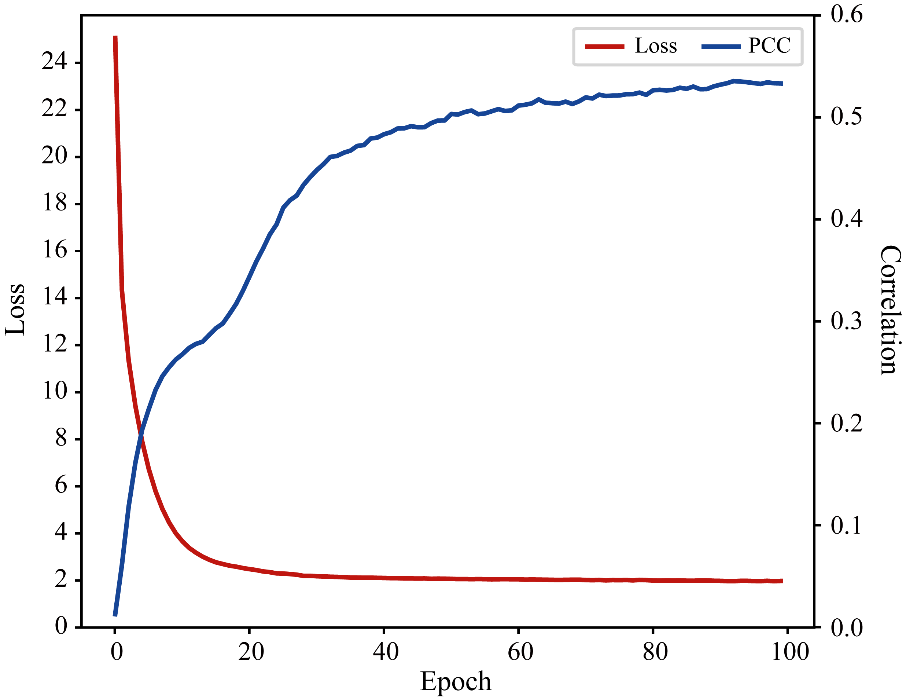


**Figure S5.** Performance of the feature generation model. The loss and Pearson correlation coefficients along epochs in cross-validation.

**Table S8.** The average performance of the feature generation model tested by splitting the dataset into separate train-test sets.

|  | **MSE** | **Pearson correlation coeffecient** | **R^2^** | **Concordance index (C-index)** |
| --- | --- | --- | --- | --- |
| Top 5% | 0.855($\pm$0.002) | 0.542($\pm$0.001) | 0.238($\pm$0.001) | 0.689($\pm$0.000) |
| Top 10% | 0.977($\pm$0.002) | 0.460($\pm$0.001) | 0.172($\pm$0.001) | 0.657($\pm$0.001) |
| Top 25% | 1.225($\pm$0.002) | 0.335($\pm$0.001) | 0.088($\pm$0.000) | 0.608($\pm$0.000) |
| Average | 2.591($\pm$0.010) | 0.224($\pm$0.001) | -0.088($\pm$0.003) | 0.564($\pm$0.000) |


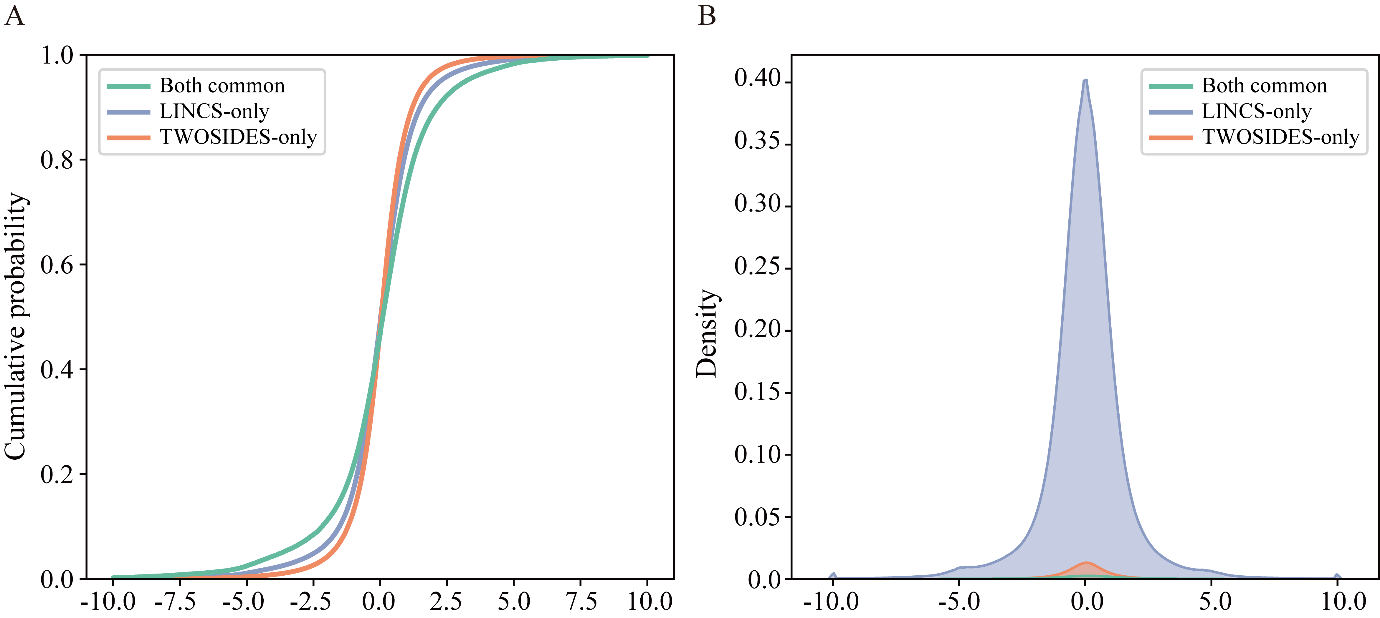


**Figure S6.** Cumulative density function (A) and probability density function (B) for each dataset value. The “both common” marker represents the overlapping compounds between LINCS and TWOSIDES datasets, and the others refer to compounds that are only in each dataset. Here, the distribution of the TWOSIDES-only dataset was made from the predicted expression values.

## **Performance of DDI prediction model**

### Robustness of DDI prediction model

We analyzed the prediction performance of the constructed DDI model. Overall, the model showed a good prediction performance – AUC of 0.889 and AUPR of 0.915. However, there was a difference in performance for each side effect type, and we checked whether this was affected by the number of data. As shown in **Figure S7**, there is no correlation between the model performance and the size of related drug pairs indicating that the model is not biased to the size of trained data.

Moreover, we analyzed the similarities between side effects. From the Unified Medical Language System (UMLS), we downloaded the hierarchical structure of diseases. Then, 463,203 pairwise similarities of side effects were calculated using the lowest common ancestor algorithm on trees. If both side effects were found in multiple trees, then the maximum similarity was considered, and if both were not found in any of the trees, then the similarity is zero. We extracted the list of side effects at top k side effect similarities to confirm whether closely related side effects showed better performance. The result shows that the similarity of side effects are not related to model performance, and shows consistent performance (**Figure S8.A**). Also, 838 out of 963 side effects were found in one hierarchical tree which enabled to compare the performance in depth-wise. Depths from the root node of side effect terms were obtained, and categorized by depth. The depth in the UMLS tree indicates the broader and narrower concepts, therefore, broader side effects may have more relations with other side effects. The **Figure S8.B** also shows consistent performance regardless of depth. These analyses indicate that the model is not biased to side effect frequency nor inner-connections.


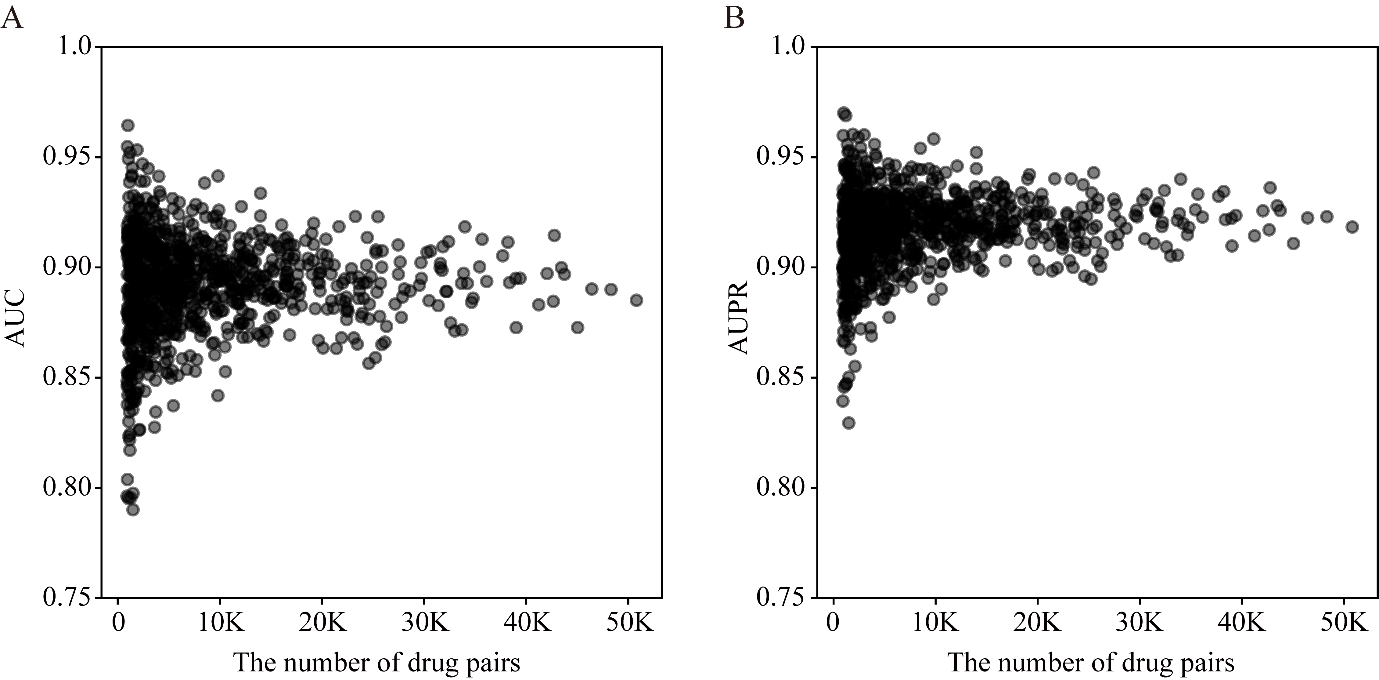


**Figure S7.** The correlations between AUC(A) or AUPR(B) with the frequency of side effect. Each point represents each side effect type. The x axis shows the number of drug pairs for each side effect which indicates the frequency.


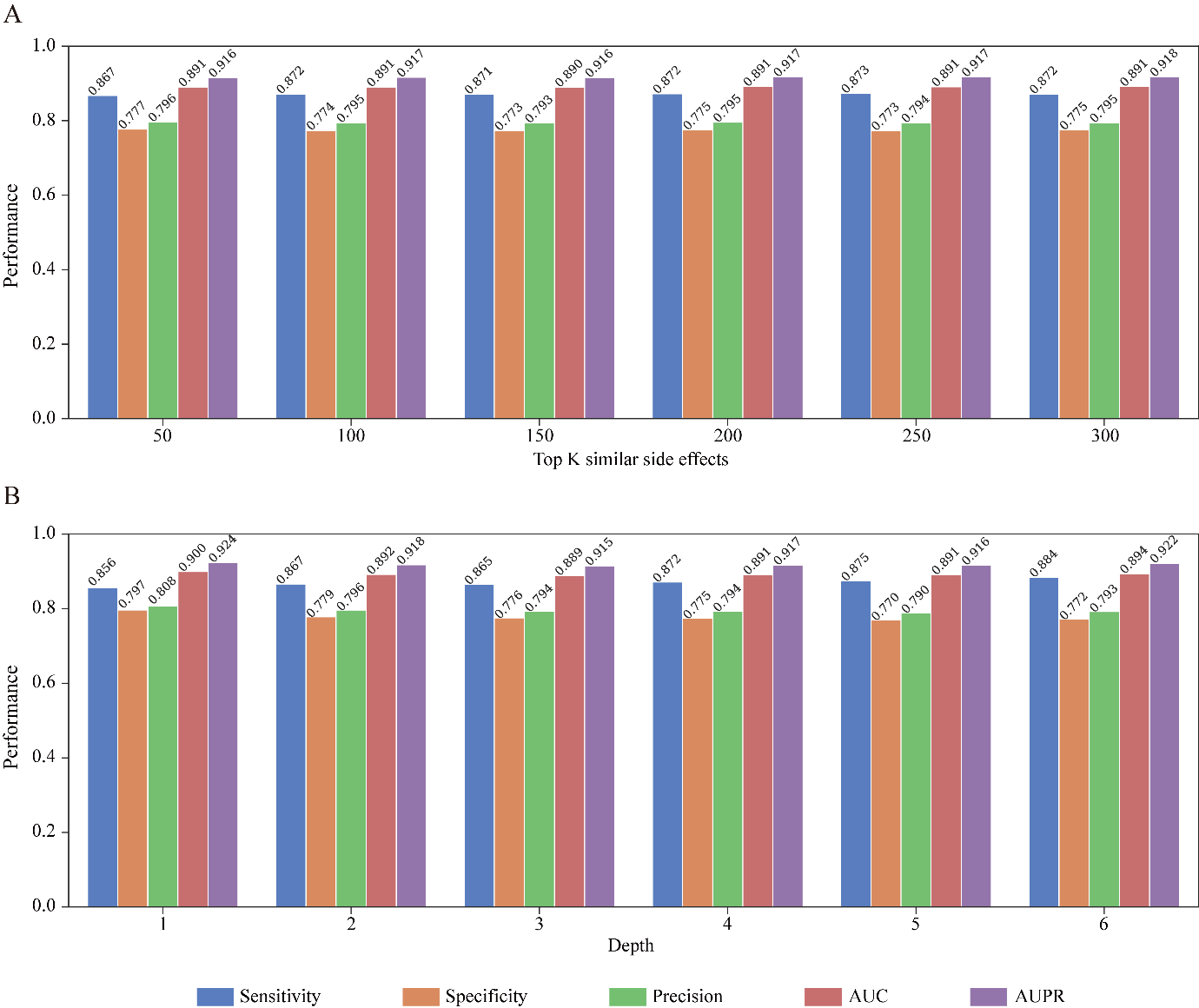


**Figure S8.** A.The performance comparison in terms of side effect similarities. The subset of side effects were selected as top k similar side effects. The similarity represents how close the side effects are located in hierarchical trees. B. The performance comparison in terms of side effect hierarchy. Side effects were categorized by depth.

The last analysis on prediction performance in terms of side effects is the relationship between single drug side effects and polypharmacy side effects. We preprocessed and integrated single drug side effect data from SIDER, OFFSIDES, and FAERS. Then, the side effect labels of each drug and interactions were compared. **Figure S9** shows the proportion of each type of triplet. 63% of triplets were not related to any reported side effects solely. It was confirmed that the drugs involved in 37% of the total triplets have single side effects. Either drug in drug pairs in 32.84% of triplets have a single drug side effect, especially 30.66% showed the same labels as DDIs. There are 4.14% of triplets whose drugs both have single drug side effects.


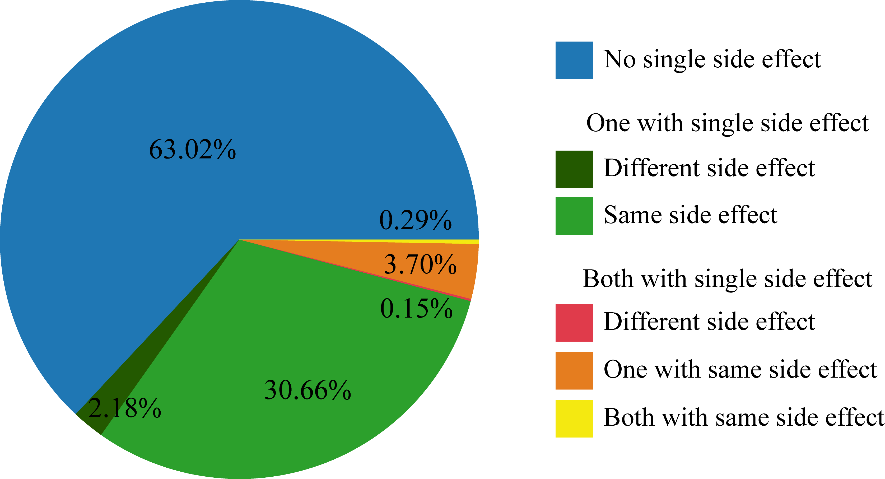


**Figure S9.** The proportion of triplets having single drug side effect in each condition.

Because the training dataset was originally from FAERS, it is hard to discriminate whether a polypharmacy side effect is induced by either drug when involved drugs are known to have the same side effect. Therefore, we confirmed the model in terms of performance by comparing the predicted scores of a drug pair and either drug, assuming that if a reported polypharmacy side effect is the result of a single drug side effect, the model may predict well with information of one drug. Meanwhile, we confirmed whether the constructed model can predict drug pairs as same when the order of drugs are switched. First, we compared the predicted scores of the original pairs (drug1-drug2) and reversed pairs (drug2-drug1). As shown in **Figure S10.A**, the model predicted both pairs almost identically. On the other hand, the predicted scores were compared with the ones from backgrounds. The backgrounds were set to be pairs of identical drugs from each position – drug1-drug1 and drug2-drug2 – predicting polypharmacy side effects with one drug. We estimated both Pearson correlation coeffictions and the coefficient of determination (*R^2^*) between original and background prediction scores, and the results indicate that the prediction scores are not influenced by one side (**Figure S10.B** and **C**).


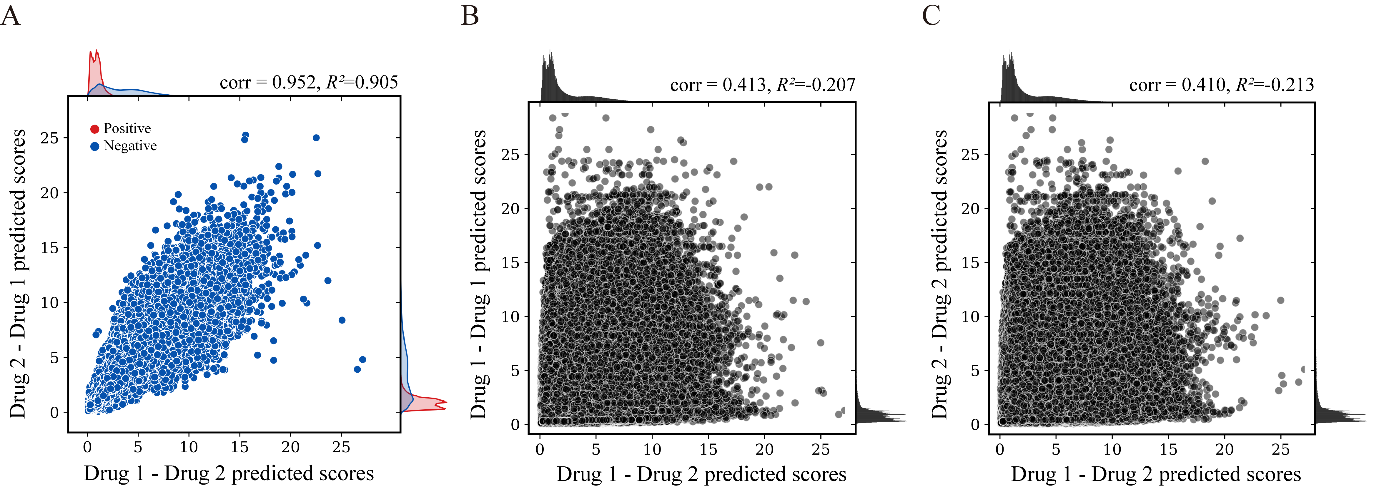


**Figure S10.** A.The correlations between predicted scores of original pairs and reversed pairs. B, C. The scatter plots of predicted scores of original pairs and backgrounds (drug1-drug1 and drug2-drug2).

### Impact of weighted features in DDI prediction

We also confirmed that the predicted expression features contributed to the model performance despite of its relatively large dimension. In general, large size of features can induce overfitting. However, to analyze the significant genes of interest in interacting drug pairs, 978-dimensional features were needed. To confirm whether the model is overfitted by the feature size, we tested models with different size of features by reducing with additional dense layers. As shown in **Figure S11**, the model with original features showed the best performance despite its feature dimension in all cases. Also, the results show that the input feature dimension is not sensitive to model performance. Therefore, the use of predicted gene expressions can help model perform better as well as feature analysis.


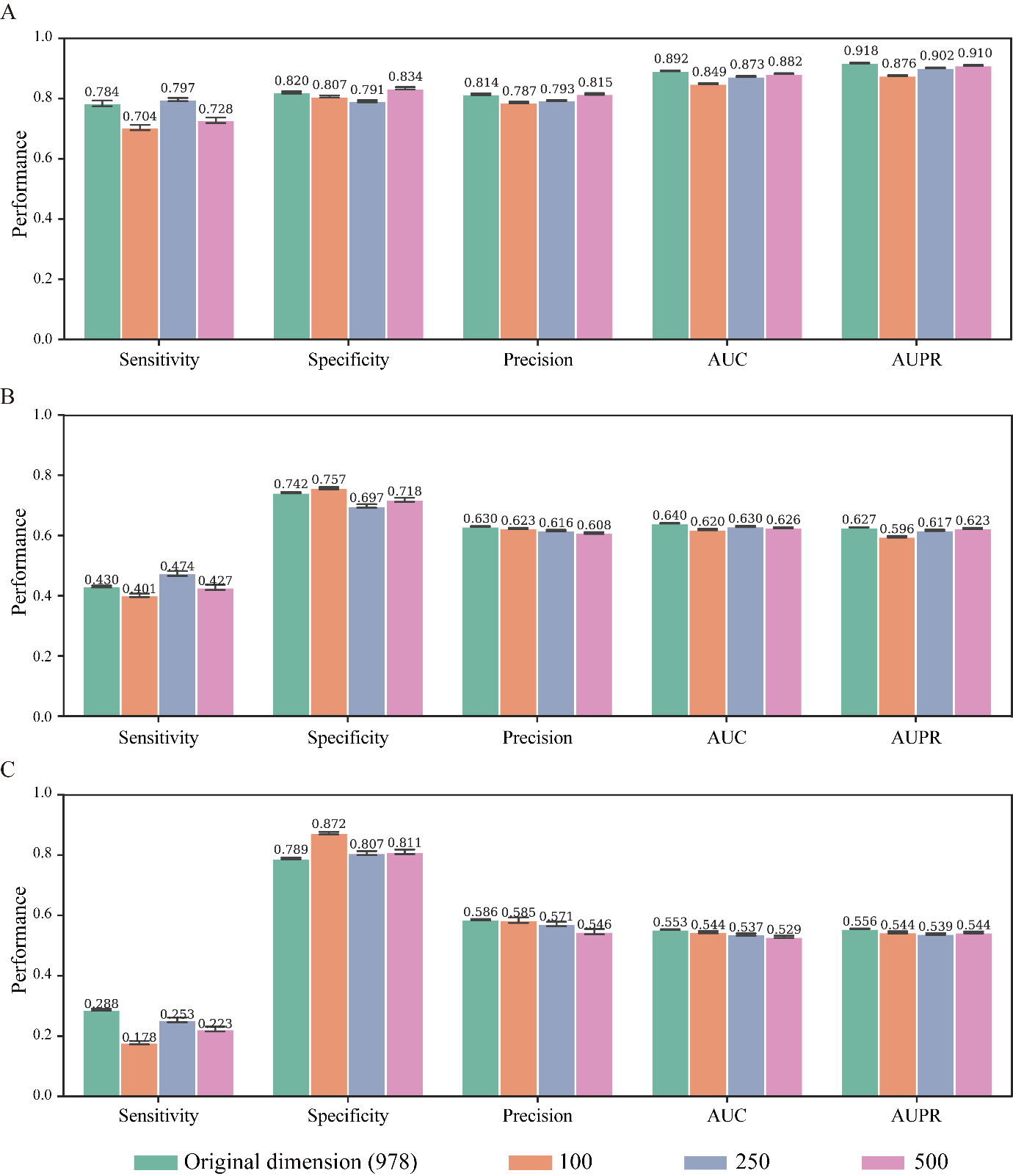


**Figure S11.** The comparison of DDI model performance according to input feature dimensions in case 1(A), case 2(B) and case 3(C). In all cases, the constructed model with predicted gene expressions resulted in the best performance in terms of AUC and AUPR.

## **Validation of predicted potential interactions in TWOSIDES**

With the prediction results from the internal test (i.e., TWOSIDES testing set), we searched for newly predicted interactions that were false positives in the DrugBank database. This validation was processed using models having different initial values used as error bars. The testing set for unseen interactions was predicted with 10 different models, and the average number of drug pairs were counted. Among total test triplets, 78,103 were predicted to have DDIs; but they were not reported in the TWOSIDES on average. The predicted triplets were validated with the DrugBank database, which also provided DDI information. The DDIs in DrugBank are described at the molecular level, unlike TWOSIDES, which has phenotypic information. To validate triplets having binary labels, we ignored side-effect types and counted drug pairs only. Finally, 2,734 drug pairs out of 19,812 were found to have drug interactions by all models (**Figure S12**) and examples of validated DDIs are listed in **Table S8**.


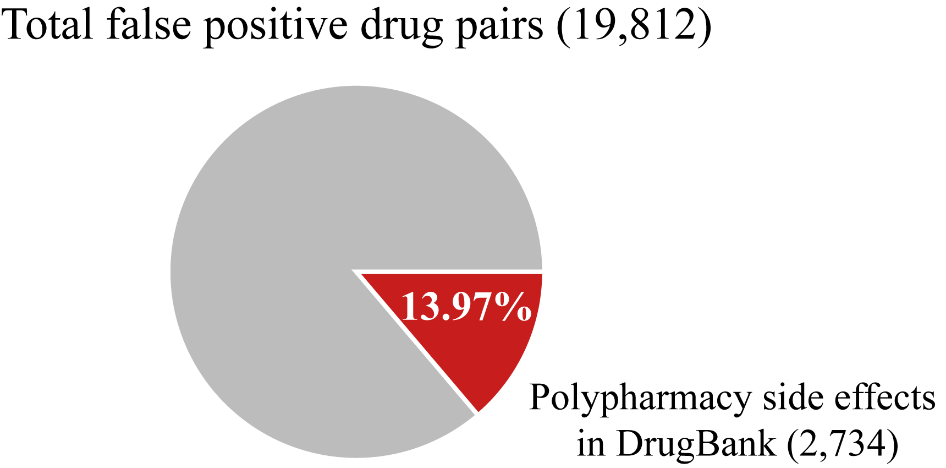


**Figure S12.** The proportion of drug pairs found in DrugBank among newly predicted polypharmacy side effects in TWOSIDES. The existence of interactions was considered, owing to the difference in description level between databases, and 13.97 % of drug pairs were searched as positives.

**Table S9.** Validation of predicted polypharmacy side effects with DrugBank

| **Drug 1** | **Drug 2** | **Side effect** | **DDI description in DrugBank** |
| --- | --- | --- | --- |
| buspirone | mirtazapine | granuloma | Buspirone may increase the serotonergic activities of Mirtazapine |
| donepezil | metoprolol | faecal incontinence | The metabolism of Donepezil can be decreased when combined with Metoprolol |
| verapamil | metoprolol | reflux esophagitis | Temazepam may decrease the excretion rate of Verapamil which could result in a higher serum level |
| cyclobenzaprine | venlafaxine | gangrene | The risk or severity of CNS depression can be increased when Venlafaxine is combined with Cyclobenzaprine |
| fluoxetine | orphenadrine | Diplopia | Fluoxetine may increase the central nervous system depressant (CNS depressant) activities of Orphenadrine |

## **Analysis on internal validation**

Alongside the best and worst performing side effects of our model, we listed the AUPR of the side effects as reported in Decagon. We compared the model performance for each side-effect type, which performed the best and the worst according to Decagon. As can be seen in **Table S9**, our model shows comparable performance in the best-performing side effects and higher performance in the worst-performing ones. Using the results in **Table 4**, our model and Decagon both show high performance, whereas each model has different strengths regarding the side-effect type.

**Table S10.** Comparison of Decagon’s best/worst performing side effects

| **Best performing side effects** | **Decagon**  **AUPR** | **Our model**  **AUPR** | **Worst performing side effects** | **Decagon**  **AUPR** | **Our model**  **AUPR** |
| --- | --- | --- | --- | --- | --- |
| Mumps | 0.964 | 0.933 | Bleeding | 0.679 | 0.895 |
| Carbuncle | 0.949 | 0.900 | body temperature increased | 0.680 | 0.910 |
| Coccydynia | 0.943 | 0.922 | Emesis | 0.693 | 0.914 |
| Tympanic membrane perforation | 0.941 | 0.922 | Renal disorder | 0.694 | 0.916 |
| Dyshidrosis | 0.938 | 0.899 | Leucopenia | 0.695 | 0.919 |
| Spondylosis | 0.929 | 0.922 | Diarrhea | 0.705 | 0.917 |
| Schizoaffective disorder | 0.919 | 0.939 | Icterus | 0.707 | 0.900 |
| Breast dysplasia | 0.918 | 0.923 | Nausea | 0.711 | 0.911 |
| Ganglion | 0.909 | 0.929 | Itch | 0.712 | 0.911 |
| Uterine polyp | 0.908 | 0.914 | Anemia | 0.712 | 0.923 |

## **External validation**

We further evaluated the model with an external dataset using a chronological validation method. The drug pairs from the old version of the dataset were predicted and validated using the new version to confirm whether the model can capture potential interactions. Here, we used DrugBank v.5.0.0 as an older version to include 33,497 positive and 603,259 negative pairs from 1,129 drugs. We noted negative pairs; however, these were unknown interactions that indicate there may be potential interactions. To confirm, we incorporated DrugBank v.5.1.7 as a new dataset. All pairs from v.5.0.0 were predicted with 963 side effects and labeled with a threshold of 65. The model prediction resulted in 9,543 true positives and 137,201 false positives. Then, all predicted positives were examined to see whether their labels changed in the new version. Among all false positives, 53,322 drug pairs were found to be positive in v.5.1.7. We also confirmed the following results with various side-effect cutoffs regarding whether the model caught true positives well. As shown in **Figure S13**, the proportions of validated drug pairs did not change much regardless of cutoffs, although the corresponding numbers of drug pairs changed. Moreover, we analyzed which cases the drug pair corresponded to. All drug pairs were categorized into unseen interactions, one seen drug, and both unseen drugs cases by confirming overlapping drugs with TWOSIDES. As shown in **Figure S14**, most drug pairs belonged to cases 2 and 3. This may explain how the model showed relatively low sensitivity because the DrugBank dataset includes one-unseen and both-unseen drug cases. The model also resulted in low precision induced by many false positives. However, the precision was dramatically increased in the new version. These results show that our model can capture potential interactions while predicting interactions with new drugs.


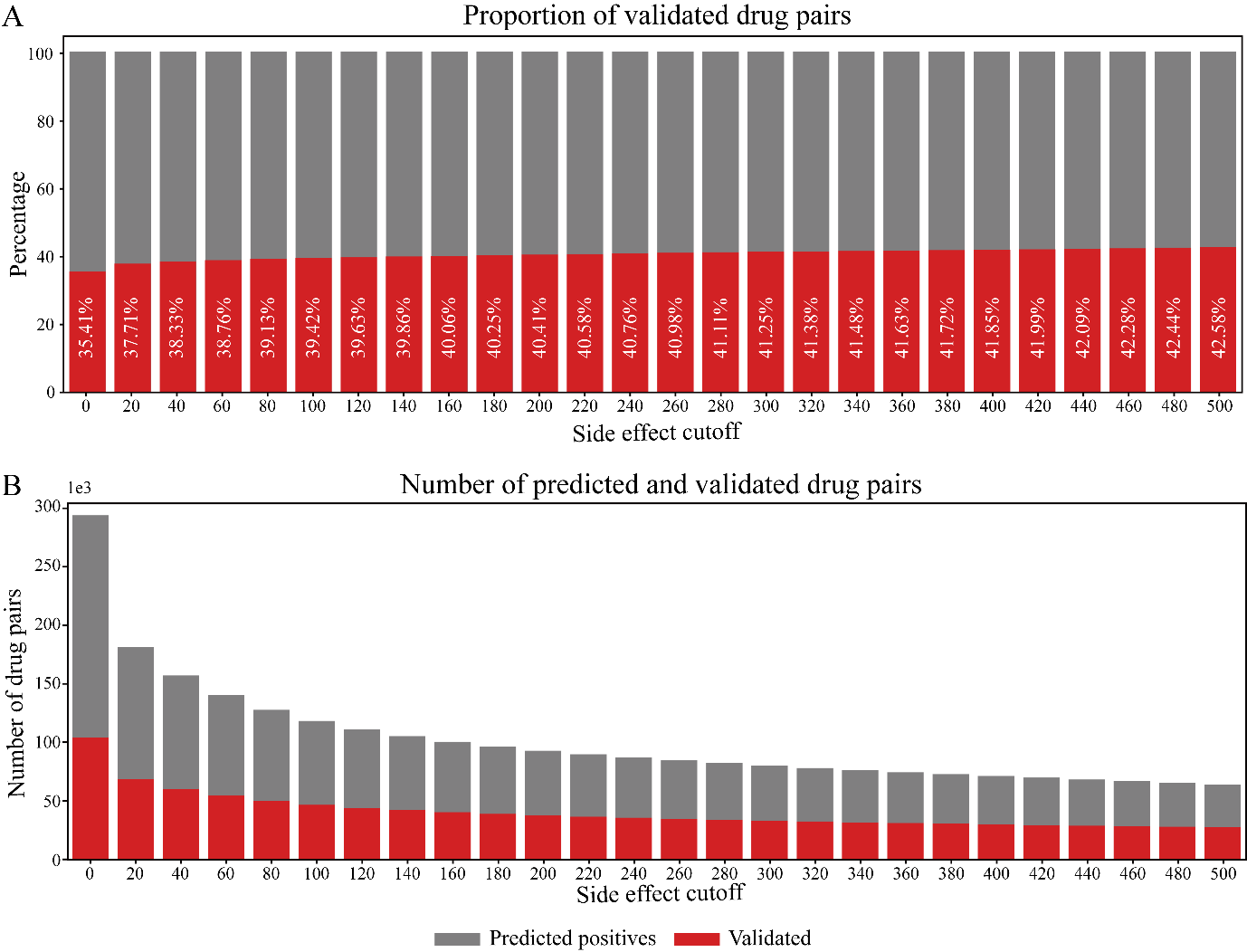


**Figure S13.** A. Proportion of validated drug pairs (hits) for each side effect cutoff. B. Number of predicted (false positives) and validated (hits) drug pairs for each side effect cutoff.


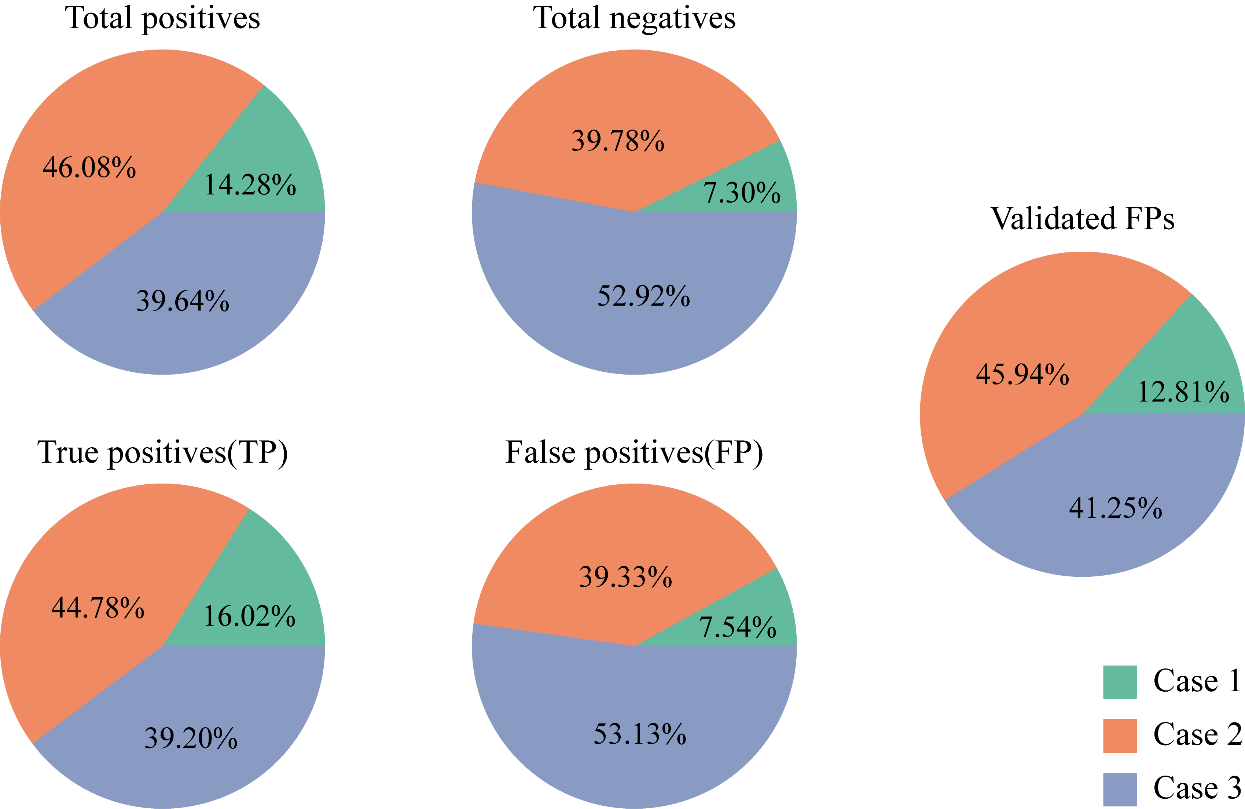


**Figure S14.** The proportion of cases of drug pairs in the DrugBank dataset. A relatively small proportion of drug pairs belongs to the case of the unseen interaction.

## **Feature analysis**

The key to predicting DDI is being able to catch different responses when one drug is taken with another. However, how the drug responses will change is not clearly known. We used the gating mechanism to reflect that the drug was the same, but the response of the drug can differ, depending on the drugs taken together. The input of the GLU included latent representations of two drugs, and the sigmoid function was used as the activation function, giving a value between zero and one for each feature. The calculated vector was multiplied by the feature before activation in an element-wise manner, transforming a drug feature into an interacting drug-relevant feature. Given drugs A and B, the GLU module operated as $drug_{A}\otimes\sigma([drug_{A,}drug_{B}])$ to process drug A’s feature. Here, the output of sigmoid function varied, depending on the paired drugs, showing that even the same drug may have different reactions, depending on the drugs taken together. For further analysis, we selected the compound of interest and observed changed features against each paired compound. We first chose an cyclophosphamide compound, which is an anticancer drug. Then, cyclophosphamide and the other paired compounds were put into the model to extract intermediate features after the GLU and to analyze the feature change of the selected compound. Then, hierarchical clustering was carried out on features and compound samples by correlation. The heatmap of feature values can be found in **Figure 5A**, of which each row represents the same drug in a different pair, and each column indicates genes. The total number of positive DDI pairs was 318, and the clustering number was set to four to see the boundary wherein the pattern was divided. Despite most are clustered into the major cluster, we can now see the distinct features between clusters. **Figure S15** shows how far apart the latent representations were from each other: all diverged from the original point. Owing to different features, which depend on the drugs taken together (even when it is the same), we assumed that each cluster of drug pairs had different side-effect patterns and characteristics. We first examined the therapeutic classes of each drug to see whether any specific class is enriched to each cluster. The anatomical therapeutic chemical (ATC) code is a unique code that categorizes a drug according to its purpose or system it works defined by the World Health Organization (WHO). While ATC codes consist of five levels, the second level divides the main group into therapeutic or pharmacological subgroups. We categorized the drugs using this information. To select representative classes for each cluster, the enrichment test was conducted. For each code, the odds ratio was calculated as the proportion of drugs with the code in a cluster to the proportion of drugs with the code in total. During this process, the codes appeared with less than three drugs were discarded to avoid them to be selected as top enriched codes due to small samples. As shown in **Figure 5A**, the top enriched ATC codes are illustrated which resulted from tests with 3, 22, 12, and 265 drugs found in each cluster. The total list can be found in **Additional file 8**. For each cluster, we then extracted the side effects that were most frequent by calculating the frequency odds ratio within the cluster vs. the total dataset. Because each cluster presents a different feature pattern, different side effects occurred (see **Table S10**). All detected side effects were uncommon, solely indicating that they are drug-specific. We believe that selecting significant genes and differentiating single drug features in terms of drug pairs improves prediction ability and can help further analyze how and when data sparsity is handled.

**Table S11**. Top frequent side effects of each cluster in cyclophosphamide.

|  | Rank 1 | Rank 2 | Rank 3 | Rank 4 | Rank 5 |
| --- | --- | --- | --- | --- | --- |
| Cluster 1 | neonatal respiratory distress syndrome | hypovitaminosis | ventricular septal defect | platelet disorder | connective tissue disease |
| Cluster 2 | xerosis | corneal disorder | mycosis fungoides | superior  vena cava syndrome | glioblastoma multiforme |
| Cluster 3 | colonic obstruction | atypical mycobacterial infection | hypogammaglobulinaemia | superior  vena cava syndrome | toxic shock |
| Cluster 4 | parotid gland enlargement | xerosis | chicken pox | aspergillosis | CMV infection |


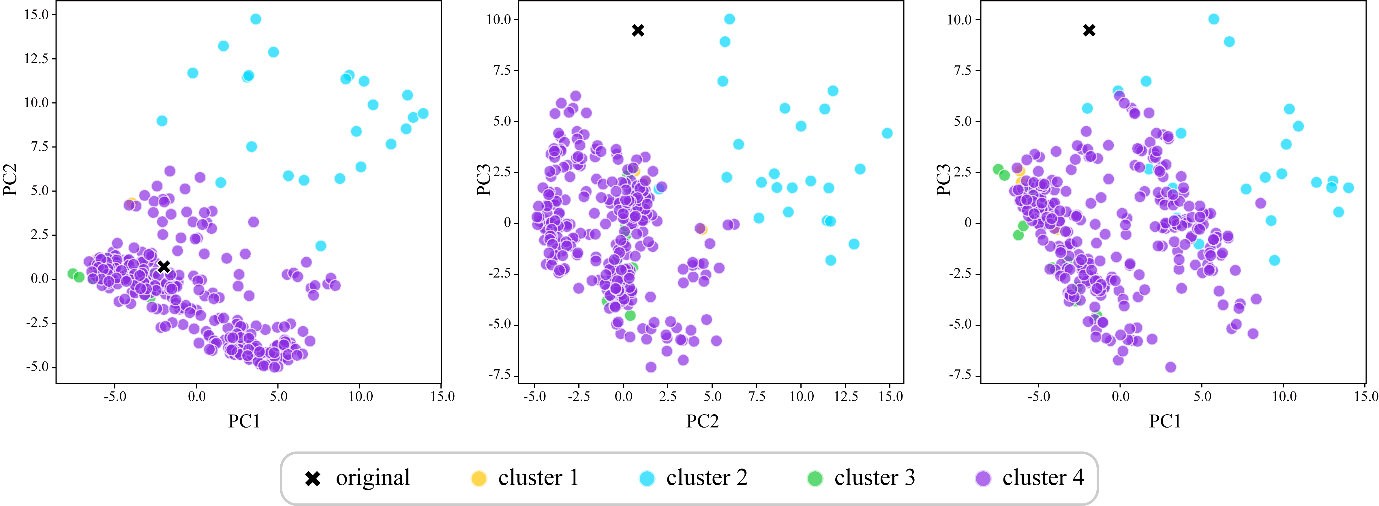


**Figure S15.** PCA plots of latent representations of cyclophosphamides with each paired drugs
